# Energy Constraints Determine the Selection of Reaching Movement Trajectories in Macaque Monkeys

**DOI:** 10.1523/ENEURO.0385-24.2025

**Published:** 2025-09-30

**Authors:** Shrabasti Jana, Lucio Condro, Frédéric V. Barthélemy, Junji Ito, Alexa Riehle, Sonja Grün, Thomas Brochier

**Affiliations:** ^1^Institut de Neurosciences de la Timone (INT), UMR 7289, CNRS, Aix-Marseille Université, Marseille 13005, France; ^2^Institute for Advanced Simulation (IAS-6) and JARA-Institute Brain Structure-Function Relationships (INM-10), Jülich Research Centre, Jülich 52425, Germany; ^3^Theoretical Systems Neurobiology, RWTH Aachen University, Aachen 52056, Germany

**Keywords:** arm movement, computational modeling, kinetic energy, macaque monkey, psychophysics, trajectory selection

## Abstract

Reaching movements, while seemingly simple, involve complex motor control mechanisms that select specific trajectories from infinite possibilities. Despite inherent variability in volitional movements, both humans and monkeys frequently exhibit stereotyped trajectories. The literature has offered numerous explanations for invariant trajectory shapes, including a common planning space in hand space or joint space, as well as factors like kinetic energy (KE) minimization and sensory feedback. However, since most studies have relied on single-session data, crucial insights into the motor principles guiding trajectory selection and their evolution through extended practice remain underexplored. This study fills this gap by investigating how specific trajectories are selected and evolve with practice across multiple sessions, using data from two rhesus monkeys (one male, one female) performing a reaching task in a biomechanically constrained 2D setup. Our behavioral study challenges the idea of a common planning space, revealing instead a significant influence of KE on trajectory shapes. Through a novel biomechanical modeling, we quantified KE for a wide range of trajectory shapes. We discovered that trajectory selection and evolution are not simply about minimizing KE or achieving straight paths. Instead, the monkeys’ motor systems appear to prioritize maintaining a “safe KE range,” where slight changes in trajectory shapes have minimal impact on energy expenditure. These findings provide new insights into the adaptive motor control strategies, suggesting that trajectory selection involves balancing energy efficiency and flexibility. Our study enhances the understanding of trajectory selection principles, with implications for rehabilitation strategies, robotics, and broader study of motor control mechanisms.

## Significance Statement

This study provides new insights into motor control by analyzing and modeling monkey behavior, revealing that kinetic energy (KE) significantly influences trajectory shape. Our findings challenge the conventional views that trajectory selection primarily aims to maximize straightness or minimize KE. Instead, our analyses show that the motor system seeks to maintain a “safe KE range,” where small trajectory differences do not significantly impact energy expenditure. We reach this conclusion through a novel biomechanical modeling approach, which quantifies KE across a wide range of trajectory shapes for specific movements. By combining behavioral analysis with modeling, we demonstrate that trajectory selection balances efficiency and flexibility, offering valuable implications for developing rehabilitation strategies and robotic assistive devices that align with natural movement principles.

## Introduction

Ever pondered about the path your hand takes when reaching for your morning coffee? Although theoretically infinite trajectories are possible for such movements, our motor system seamlessly selects a unique trajectory for each motor goal. Studying these trajectories can unveil key insights into motor control principles.

In 1967, Bernstein first highlighted the inherent variability of volitional movements, stating “practice is a particular type of repetition without repetition” (Bernstein, 1967). This means two trajectories are never the same, even with identical goals and starting conditions. Despite this variability, we often use stereotyped trajectories with invariant features (Morasso, 1981, 1983; Abend et al., 1982). Trajectory shape is one such feature that has been extensively studied.

The invariant trajectory shape is thought to arise from a common planning space for all movements (Van Thiel et al., 1998). Two primary contenders for such a planning space are the extrinsic hand space and the intrinsic joint space.

Numerous studies (Morasso, 1981; Abend et al., 1982; Hogan, 1984; Atkeson and Hollerbach, 1985; Flash and Hogan, 1985) indicate that reaching movements are generally straight in hand space, suggesting it as the planning space. Flash and Hogan (1985) proposed the minimum jerk theory, which posits that the motor system aims to produce smooth, straight movements in hand space.

Other studies (Atkeson and Hollerbach, 1985; Hogan and Flash, 1987; Haggard and Richardson, 1996) show that hand trajectories often deviate from straight lines and vary with movement direction and location. These deviations could stem from joint-level control due to varying constraints on joints at different locations. Rosenbaum et al. (1995) propose that joint space could be the planning space, suggesting that straight trajectory in joint space, with coordinated joint movements, is a crucial motor principle. Due to the nonlinear relationship between joint space and hand space, straight joint space trajectories can result in curved hand paths (Hogan et al., 1987). This aligns with the minimum torque-change model (Uno et al., 1989), which predicts curved hand paths based on joint torques (Uno et al., 1989). A force-field study further supports joint space as the planning space, demonstrating that adaptation learning transfers more easily in joint space than in hand space (Malfait et al., 2002).

Altogether, opinions vary widely on whether hand space or joint space serves as a common movement planning space. Cruse and Brüwer (1987) propose a compromise between hand space and joint space, suggesting no single planning space applies to all movements. Van Thiel et al. (1998) similarly argue that task context and coordination of both spaces together shape trajectories.

More recently, Bongers and Zaal (2010) reported that beyond planning space, factors like surface shape and friction influence trajectory curvature by altering movement resistance, which in turn interacts with biomechanical forces or kinetic energy (KE) in shaping trajectories. KE minimization is also suggested by many others (Hatze and Buys, 1977; Nelson, 1983; Cruse, 1986; Soechting et al., 1995; Alexander, 1997; Kashima and Isurugi, 1998; Engelbrecht, 2001; Todorov and Jordan, 2002; Todorov, 2004) and combines the effects of many biomechanical factors such as power, torque, etc.

However, a force-field task demonstrated that even when low-energy paths were curved, subjects preferred straight hand paths, challenging the notion of energy minimization as a universal principle (Kistemaker et al., 2010). This could mean that visual feedback in this case override energy considerations, leading to visually straight paths. Vaidyanathan et al. (2020) also show that the absence of visual feedback results in curved hand trajectories.

The literature thus provides various biomechanical and sensory explanations for trajectory selection. However, it lacks the crucial perspective of how practice influences trajectory selection and evolution. Additionally, past studies have predominantly used standardized tasks with humans, generally featuring only radially organized trajectories and similar movement amplitudes.

In this study, we investigate how specific trajectories for a variety of movement configurations are chosen and how they evolve with practice across multiple sessions for two monkeys. Our analyses aim to determine whether trajectory selection and evolution are driven by a common planning space or other motor control rules.

## Materials and Methods

### Subjects

Two rhesus monkeys (Monkey E, female, ∼6 kg; Monkey J, male, ∼9.5 kg) participated in the current study where they performed arm-reaching movements using the KINARM exoskeleton robotic device (Scott, 1999). All experimental procedures were approved by the local ethical committee (C2EA 71; authorization Apafis#13894-2018030217116218v4) and conformed to the European and French government regulations.

### Experimental and recording setup

**Figure 1.**
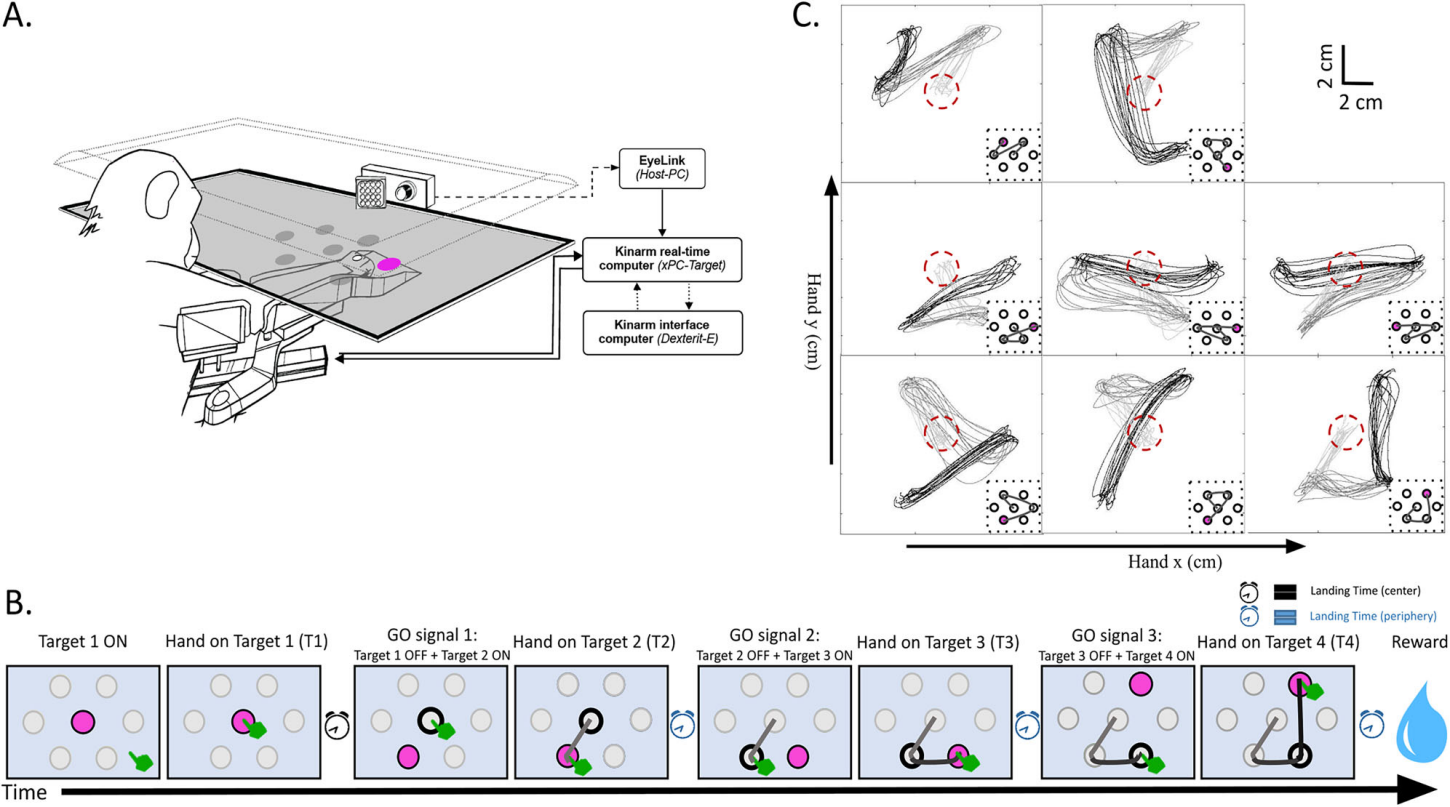
Experimental paradigm: ***A***, Experimental setup, Monkeys performed the task while seated in a primate chair with their right arm fixed in a robotic exoskeleton, constrained in a horizontal 2D plane. Visual cues appeared on a horizontal screen, guiding the monkey to move the hand-feedback cursor for successful reaching movements (adapted and modified from De Haan et al., 2018). ***B***, The task, The schematic shows the sequence of events in a successful trial. Pink circles indicate the active target. A successful trial consists of three submovements (light gray, first submovement; dark gray, second submovement; black, third submovement). ***C***, Movement sequences, Trajectories for each movement sequence from an example session (light gray, first submovement; dark gray, second submovement; black, third submovement). Red dashed circles indicate the central target position. Insets show the target sequence with the final target position in pink.

In this study, the monkeys were trained to use a robotic interface (KINARM, BKIN Technologies) to perform arm movements toward visual targets in the horizontal plane, as shown in the schematic (Fig. 1*A*). The right arm and forearm were supported by an articulated exoskeleton allowing planar shoulder and elbow motion and independent monitoring of the two joint motions. The opposite nonworking left arm was restrained in a semiflexed position to avoid motor interference. This apparatus was coupled with a virtual-reality system composed of a horizontal semireflective mirror at chin height that masked direct arm view and a computer screen placed horizontally above the mirror and facing downward. This screen allowed projection of white visual targets (0.65 cm in diameter) and a cursor (white hollow circle, 0.3 cm in radius) on the mirror indicating the position of the monkey's hand in the workspace at any time. The monkeys were not head-fixed, but the movement of the head was minimized using a custom-designed 3D–printed mask, which also included a reward tube. The recordings of the behavioral data used in the current study were performed simultaneously with the recordings of electrophysiological data from five cortical areas, namely, areas V1, V2 in the visual cortex, areas 7A, DP in the parietal cortex, and M1/PMd in the motor cortex using Utah arrays. Eye movements were tracked using an EyeLink eye tracker, as the monkeys performed the visuomotor task.

### Task and training

The monkeys were water restricted in their home cage and trained to perform a sequential reaching task in the laboratory to obtain water rewards.

The monkeys’ goal was to perform arm movements to reach a sequence of three visual targets located at one of the six vertices of a hexagon presented on the horizontal screen (Fig. 1*B*). Each trial was initiated by the appearance of a circular target (0.65 cm diameter) at the center of the hexagon on the screen. The monkeys had to reach this starting point by moving the hand cursor and to hold this position for 250 ms before the central target disappeared and one of the six peripheral targets was illuminated. The central target extinction also instructed the monkeys to reach the active target position and to hold this position for a fixed duration (150–200 ms). At the end of the holding period, the active target was turned off and the next target in the sequence was illuminated. This event served as the GO signal for the next reaching movement. The same sequence was repeated until the monkeys reached a sequence of three peripheral targets. The distance between the centers of the central target and the top (or, equally, the bottom) targets of the hexagon was 3.6 and 4 cm for Monkey E and Monkey J, respectively. The distance between the central target and the right (or, equally, the left) target was 4.5 and 5 cm for Monkey E and Monkey J, respectively. Successful trials were rewarded with a drop of water. If the monkeys failed to reach one of the targets or to hold the target positions long enough, the trial was aborted, and a new trial started with the same target sequence as the preceding failed trial. In addition, the trial was also aborted if the movement time between two targets exceeded 1.5 s. To facilitate the task and to encourage fast and natural movement trajectories, the constraints on the movement precision were reduced by invisibly extending each target hitting zone to 1 cm radius (logical radius) around the target center.

The task combined eight possible movement sequences (Fig. 1*C*, insets), which differed in the order in which the three next peripheral targets appeared. These eight different movement sequences spanned a wide variety of arm movements that had unique combinations of amplitudes, arm geometries, and movement directions. We randomized the occurrence of these eight movement sequences in blocks of 40 trials (five trials per movement sequence). A complete session consisted of three such blocks (120 trials). Depending on the motivation of the monkeys, we recorded anywhere between one and four sessions in a day, usually with no intervention from the experimenters during the intersession intervals.

### Datasets

In this study, we have analyzed behavioral data from 20 sessions in Monkey E and 42 sessions in Monkey J recorded over several months. Each dataset is made up of several kinematic measurements: the endpoint hand position, the shoulder and elbow joint angles, and the hand and joint movement velocities. All these movement parameters were recorded at a frequency of 1 kHz.

### Data analysis

All kinematic data were analyzed offline using custom-written scripts in MATLAB (The MathWorks).

#### Submovements

First, the kinematic measurements of each movement sequence were sectioned to separate the three submovements that make them up, each starting from one target and ending at the next target in the trial sequence. This sectioning was based on two behavioral events: the time at which the hand reached the starting target location and the time at which it reached the next target in the sequence. Submovements from different sequences but with identical start and end target locations were grouped together. This led to the reduction of the original 24 groups of submovements into 19 equivalent groups of submovements, which we consider as the fundamental submovement grouping in all the following analyses.

#### Detection of movement onset

A custom-made three–step algorithm was used to reliably detect the hand movement onsets for every submovement in a sequence. A velocity threshold was set individually for each monkey, based on visual inspection of the hand velocity traces to identify the parts of the trajectory in which the hand was moving at high velocity. To ensure that the analysis only focused on the complete movement toward the target, we then rejected all movement sections that remained above the speed threshold for a duration of <50 ms. For the remaining segments, we then used a backward approach starting from the speed threshold crossing event to identify the immediately preceding zero-crossing time point after which the acceleration became positive. This time stamp was stored as the movement onset times for each submovement trajectory.

#### Hand space and joint space trajectories

**Figure 2.**
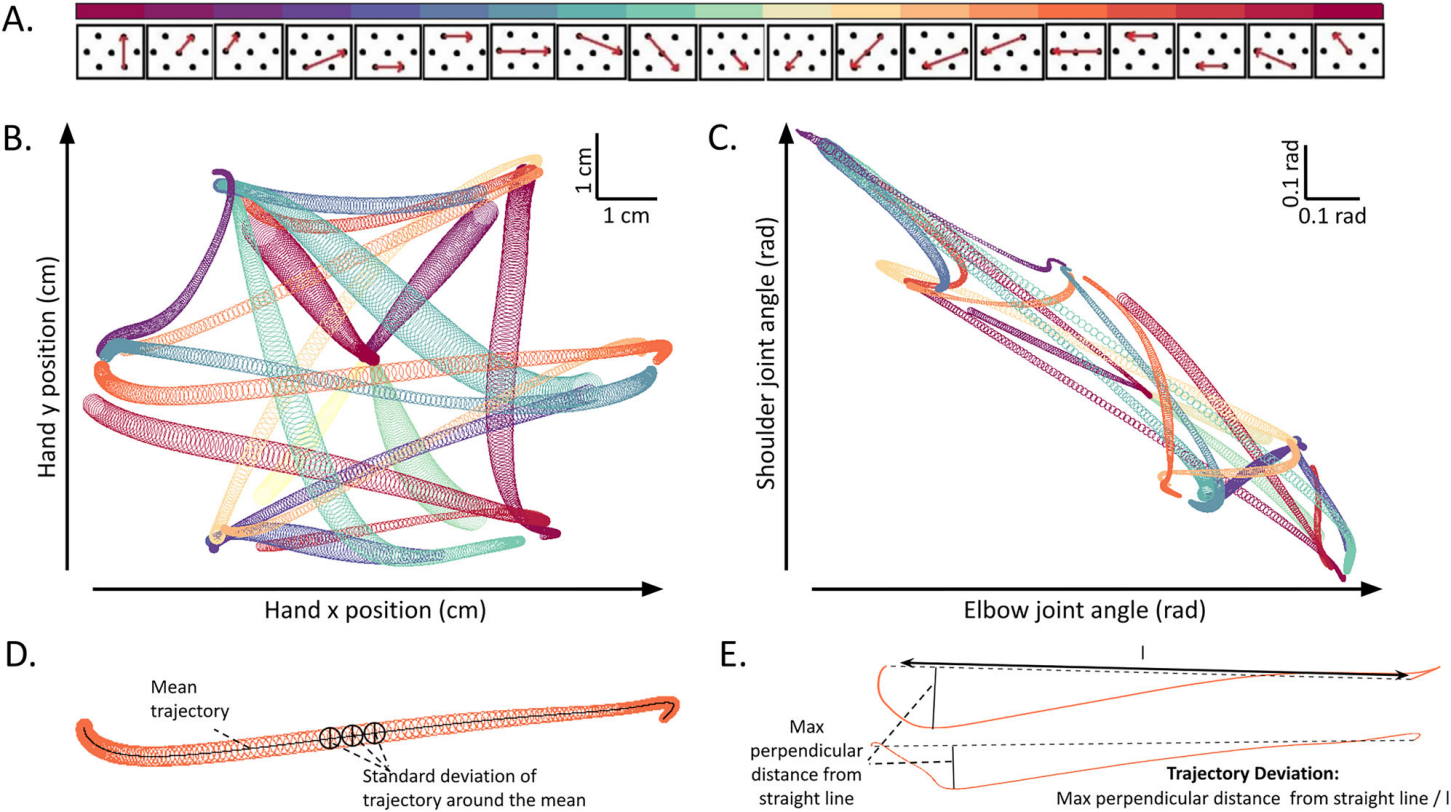
Trajectory variability and trajectory deviation in hand space and joint space: ***A***, Submovements, The panel shows all submovements with each red arrow representing a specific movement in a predefined hexagonal grid on the horizontal screen. The cyclic color scale at the top assigns a unique color to each submovement, with similar directions having similar colors. ***B***, ***C***, Hand space and joint space trajectories, Trajectory characteristics for all submovements from ***A*** are shown in ***B*** and ***C***. Circles along the length of each submovement indicate the standard deviation of trajectories for every submovement in hand space (***B***, workspace/Cartesian space) and joint space (***C***, 2***D*** space formed by shoulder vs elbow movement extent) at 10 ms intervals. Trajectories are color-coded according to A's scale. ***D***, Characterization of trajectory variability, Circles, as explained in ***B*** and ***C***, represent the spatial variability of trajectories, for an example submovement in hand space during an example session. Circles are centered on the mean trajectory (solid black line). ***E***, Characterization of trajectory deviation, Individual trajectories, for an example submovement are shown by the colored lines. For each trajectory the deviation is measured as the maximum perpendicular distance from the respective straight lines connecting the two endpoints of each trajectory, normalized by the length of these lines.

The submovement trajectories were the main focus of this study. The hand endpoint trajectory made in Cartesian space for each submovement has been referred to as the hand space trajectory (Fig. 2*B*) for that specific submovement. Similarly, the theoretical trajectory formed by the extent of shoulder and elbow movements in the angular coordinates (shoulder vs elbow; Fig. 2*C*) corresponding to that submovement has been referred to as its joint space trajectory.

#### Characterization of spatial variability

For all trajectories of each submovement, the mean trajectory was computed in both hand and joint space (Fig. 2*D*, black solid line) between the detected movement onset and the target reach. The standard deviation of all the trajectories around the mean was computed at every 10 ms interval throughout the movement. These standard deviations are represented by the diameter of the colored circles shown in Figure 2*B–D*. Since trajectories for a given submovement in a given session have variable velocity and duration, the standard deviation has been calculated for all trajectories only for the duration of the shortest trajectory.

#### Trajectory deviation

To quantify the trajectory deviation of a submovement trajectory, we first drew a straight line connecting the start and endpoint of the trajectory. Then we calculated the maximum perpendicular distance of the trajectory from this straight line, which we used as the measure of trajectory deviation for this submovement trajectory (Fig. 2*E*). This procedure was carried out for all submovement trajectories in both hand and joint space, yielding the measure of trajectory deviation in each space for each submovement trajectory.

#### Evolution of trajectory deviation

**Figure 3.**
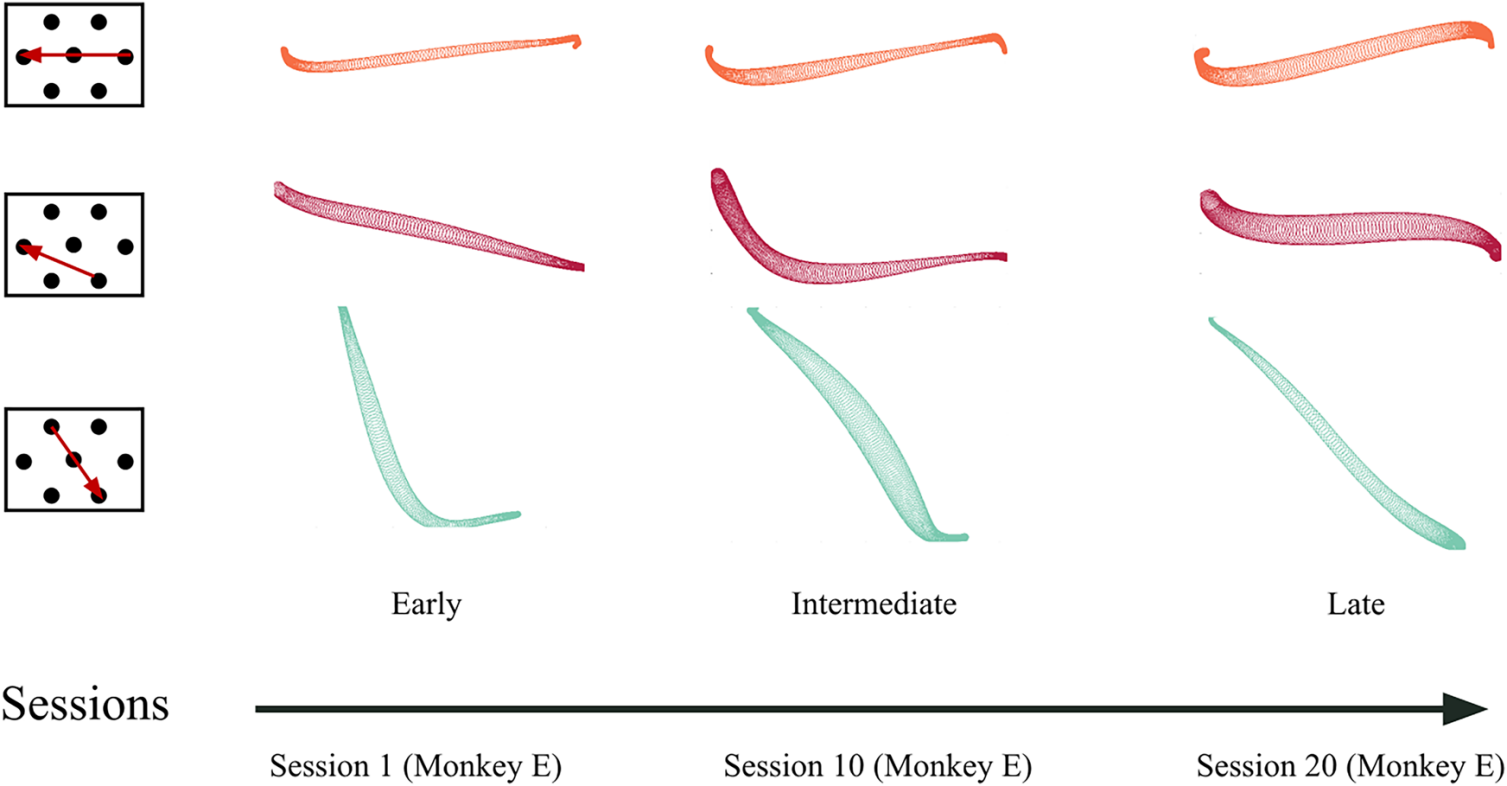
Evolution of trajectories across sessions, The leftmost column shows submovement configurations, with arrow tails indicating initial targets and arrowheads indicating final targets. The next three columns display characteristics of these submovement trajectories as explained in Figure 2 in hand space for an early session (2nd column), an intermediate session (3rd column), and a late session (4th column), for Monkey E. The examples illustrate varied trends in trajectory evolution. Submovements are color-coded as described in Figure 2*A*.

**Figure 4.**
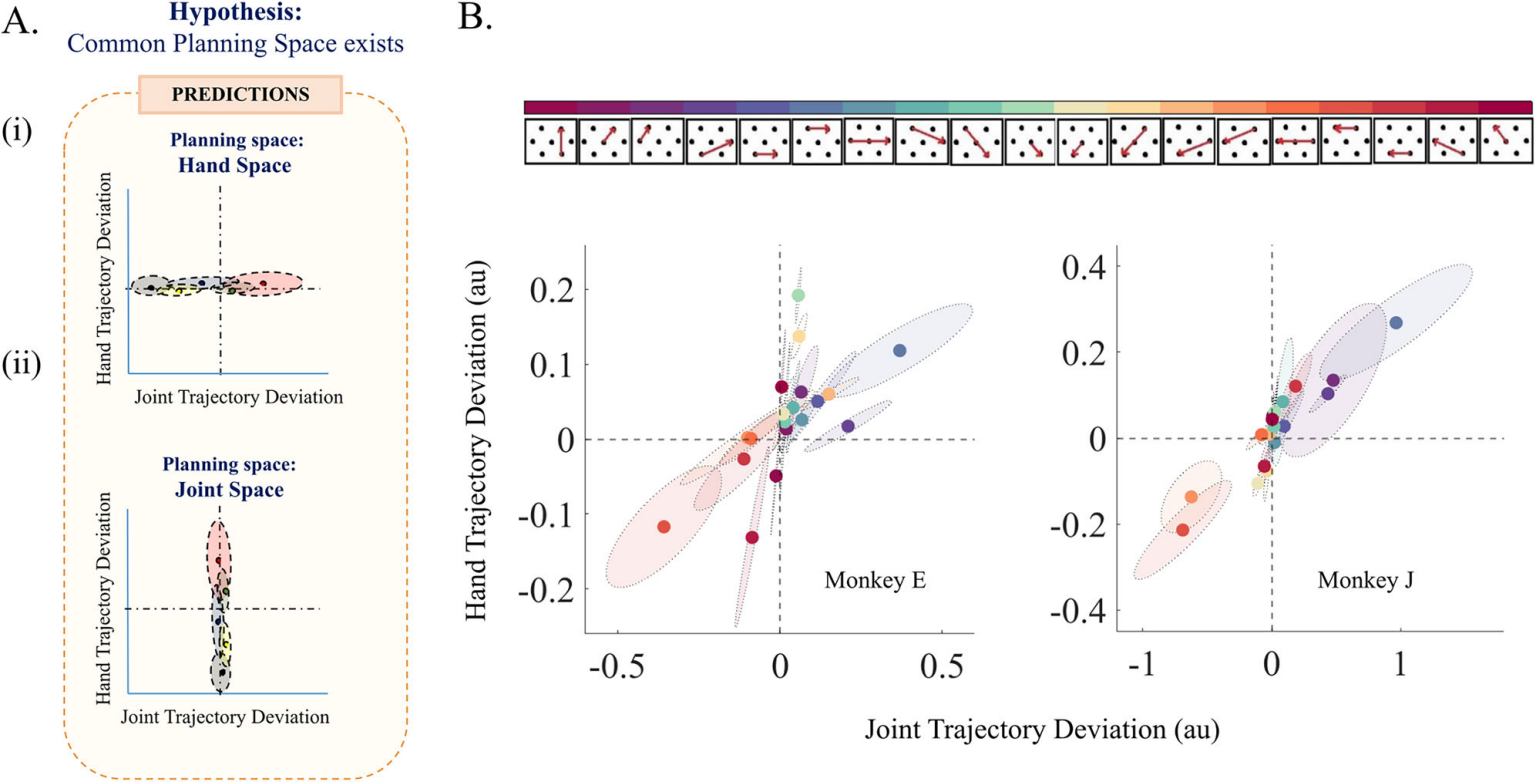
No evidence for a universal movement planning space: ***A***, Expectation with a universal movement planning space (***i***, ***ii***), predicted low trajectory deviations in a hypothetical planning space. In (***i***) hand space and (***ii***) joint space, deviations are expected to align with the horizontal and vertical dashed lines at 0 deviations. Each shaded ellipse and filled circle represent the hypothetical spread and mean of trajectory deviations in a 2D plane comparing joint (*x*-axis) and hand (*y*-axis) deviations. ***B***, Comparison of trajectory deviations in two spaces across sessions, Color-coded by submovement (Fig. 2*A*), showing the relationship between average trajectory deviations in hand space (vertical axis) and joint space (horizontal axis) across all sessions. Ellipses represent the maximum spread (standard deviation) of deviations for each submovement across the entire training period, and filled markers represent the mean joint versus hand deviation. This across-session representation reflects the temporal evolution of submovement deviations throughout the training period, capturing any consistent directional biases. Extended Data Figure 4-1 shows the distribution of angles for the major axis of the ellipses representing variability of trajectory deviations in joint versus hand space for individual sessions. Extended Data Figure 4-2 shows how the submovements could be classified into two classes based on ***B***.

**Figure 5.**
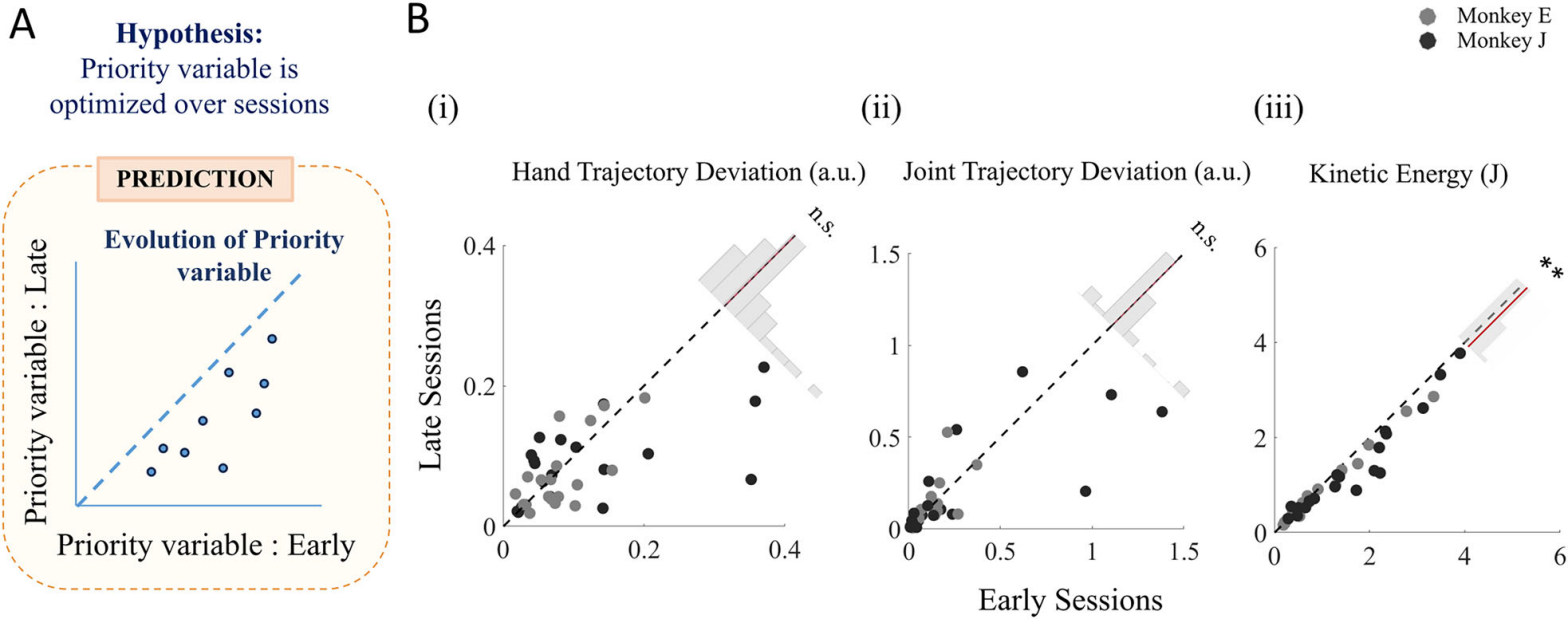
Evolution of trajectory deviations and KE through training sessions: ***A***, Prediction for evolution pattern of priority variable, Assuming there is a priority variable, optimization of which defines trajectory selection and evolution across sessions, the expectation would be that the late values of the variable will be lower than the earlier values of the variable as can be seen in this visualization. ***Bi***,***ii***, Evolution of trajectory deviation over practice, Normalized and absolute deviations of trajectories in late sessions plotted against early sessions for (***i***) hand trajectories and (***ii***) joint trajectories. Each marker (Monkey E, light gray; Monkey J, dark gray) represents the mean deviation value across sessions for a specific submovement. Insets show the distribution of the difference in trajectory deviations between early and late sessions (thick red line, mean of the distribution). No significant overall change in deviation levels across sessions (hand space, *p* = 0.8675, or joint space, *p* = 0.8790, Wilcoxon signed-rank test). Extended Data Figure 5-1 shows the change in unsigned hand and joint trajectory deviations for submovements grouped depending on whether they had high or low initial deviations (***iii***). Evolution of KE over practice, Solid markers (Monkey E, light gray; Monkey J, dark gray) represent mean KE for early versus late sessions for each submovement. The inset shows the distribution of KE differences between early and late sessions (mean indicated by the thick red line), revealing a significant global reduction in later sessions (*p* = 7.7543 × 10^−5^; Wilcoxon signed-rank test). Extended Data Figure 5-2 shows the progression of average KE for each submovement through the sessions.

To study how the trajectory shapes for the submovements evolve with practice, we first investigated them across sessions qualitatively (Fig. 3). We then quantified the variability of the measured trajectory deviations in both hand and joint spaces across all sessions (Fig. 4*B*, shaded ellipses mark the standard deviation of the deviations in the two spaces aligned along the axis representing the maximum spread of the data). In addition to the across-session analysis shown in Figure 4*B*, we quantified trial-by-trial variability within each session by fitting ellipses to the distribution of deviations for each submovement within a session. We then measured the angle between the major axis of each ellipse and the horizontal axis, thereby estimating the direction of maximal variability within a session. In Extended Data Figure 4-1, we plot these angles across all sessions separately for each of the 19 submovements, providing a direct visualization of how the direction of trial-by-trial variability is oriented relative to the hand and joint axes. To further have a quantitative measure of how the trajectory deviations evolved across sessions, we measured and compared the average deviation in hand space (Fig. 5*Bi*) and joint space (Fig. 5*Bii*) for each submovement for the first 10 (early) and last 10 (late) sessions for each monkey. This early-late session comparison allowed us to capture more stable trends in trajectory deviations, reducing the influence of session-by-session variability that could obscure broader learning effects.

10.1523/ENEURO.0385-24.2025.f4-1 Figure 4-1 **Distributions of axis angles of variability ellipse for sub-movements across sessions:** For each sub-movement (n = 19), we quantified trial-by-trial variability in hand-space and joint-space deviations within individual sessions. For each session and sub-movement, an ellipse was fitted to the distribution of deviations (similar to Figure 4B but for individual sessions), and the angle between the major axis of the ellipse and the horizontal (hand-space) axis was measured. This angle captures the direction of maximal trial-by-trial variability relative to hand and joint spaces. The distributions of these angles across sessions are plotted here separately for each sub-movement (color-coded according Figure 2). The angles for such ellipses shown in Figure 4B (captures variability of session-averaged deviations across sessions) have also been plotted for comparison and marked by a black circle for each sub-movement. **A**: Monkey E and **B**: Monkey J. Download Figure 4-1, TIF file.

10.1523/ENEURO.0385-24.2025.f4-2 Figure 4-2 **Sub-movement classes based on hand and joint deviations: A.** Sub-movement classes - Two sub-movement classes (Blue- Class 1, Orange- Class 2) were identified from Figure 4B, using kmeans clustering algorithm (distance parameter - cosine). The sub-movements classified in the two classes were identical for both the monkeys as shown in top panel. **B**. The boxplots show the KE distribution for sub-movements in classes C1 and C2, with C2 having significantly higher energy than C1 (p = 4.7862×10^-80^, Wilcoxon rank-sum test). **C.** Circles indicate the magnitude of change in KE for individuals with significant differences between early and late sessions (left - C1; right - C2), with error bars representing standard error. Download Figure 4-2, TIF file.

10.1523/ENEURO.0385-24.2025.f5-1 Figure 5-1 **Change in deviations for movements with high/low initial hand deviation:** The change in absolute trajectory deviation in both hand space (A) and joint space (B) is computed for the movements that have low early hand deviations and high early deviations. Only for movements which had high initial hand deviations, a significant reduction (p<0.05: paired sample t-test) in hand deviation was observed. Download Figure 5-1, TIF file.

10.1523/ENEURO.0385-24.2025.f5-2 Figure 5-2 **Evolution of KE through sessions: A.** For each sub-movement, the average KE for each session was calculated and plotted as a open circle. Linear regression lines were fitted to the KE values across sessions to estimate the overall trend of KE changes over time. Each panel shows the session-by-session progression of KE for one monkey. The markers and regression lines corresponding to the 19 different sub-movements are color-coded to the code presented in Figure 2. **B.** Histograms of Pearson correlation coefficients (r) from the linear regression models for each monkey summarize the consistency and directionality of KE changes across sub-movements. The overall shift of r values toward the negative side indicates a prevalent trend of KE reduction over time. Download Figure 5-2, TIF file.

#### KE

The KE for every submovement trajectory was calculated as a summation over all time points, with the energy contribution being calculated at each time point according to the following formula:

$$\mathrm{KE}=\sum_{t=1}^{n}\left(\frac{1}{2}I_{s}\omega_{s}^{2}+\frac{1}{2}I_{e}\omega_{e}^{2}\right),$$

where *n* is the number of time points and *ω_s_* and *ω_e_* are the shoulder and elbow angular velocity, respectively, recorded by the KINARM exoskeleton through every submovement trajectory during the experiment. The corresponding moments of inertia $\left(I_{s},I_{e}\right)$ were computed by approximating the two arm segments as rigid rods with weights at one end (*I* = *mL*^2^; *I* is the moment of inertia, and *m* and *L* are the mass and lengths of the rigid rod), using the approximate weights and lengths of the arm segments. The same arm lengths (0.13 and 0.23 m for upper and lower arm segments, respectively) in the exoskeleton were used as a comfortable setting for both the monkeys. Approximate weights of upper (∼220 g) and lower arm (∼90 g) segments were used for both monkeys.

#### KE evolution across sessions

Like the trajectory deviations, the evolution of KE across sessions was estimated by comparing the average KE of the submovements in the first 10 (early) sessions to the last 10 (late) sessions (Fig. 5*Biii*) to reduce the influence of session-to-session fluctuations and focus on the stable trends of KE evolutions across training sessions. However, to visualize the finer session-by-session progression of KE, we plotted the evolution of the average KE for each submovement across sessions and fitted linear regression lines to highlight the overall trends (Extended Data Fig. 5-2).

#### KE estimation model structure

**Figure 6.**
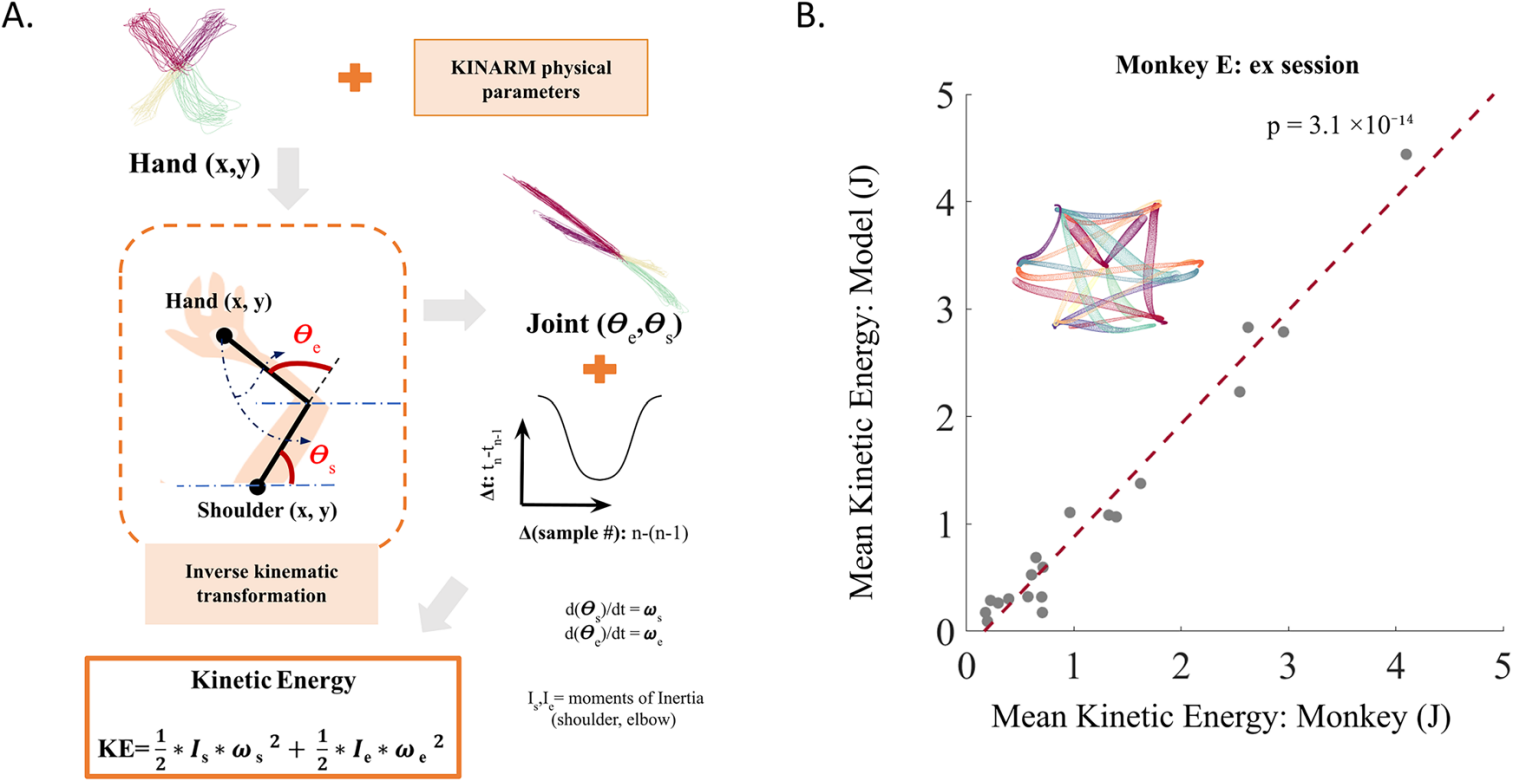
KE estimation model: ***A***, Working of KE estimation model, The schematic illustrates the major steps in estimating KE from any trajectory in either hand space or joint space. Joint space trajectories are derived from hand space trajectories through an inverse-kinematic transformation using the same biomechanical parameters as the KINARM exoskeleton. KE is then computed using these joint angles and a bell-shaped or inverted bell-shaped velocity time profile, as depicted in the KE equation at the bottom. ***B***, Validation of KE estimation model, The plot shows the mean KE for all submovements (trajectories shown in the inset) from an example session of Monkey E, compared with the mean KEs computed using the KE estimation model for the same trajectories. There is a strong correlation between the estimated and observed KE across submovements (*r* = 0.98; *p* = 3.1 × 10^−14^; Pearson's correlation).

Our modeling approach aimed to estimate the KE required for trajectories that were not naturally performed by the monkeys for each submovement. While the monkeys, in principle, could execute a wide range of trajectories, in practice, their movements were constrained to a limited range of deviation in both hand and joint space, suggesting an underlying reason for these restrictions. By modeling alternative trajectories, we sought to understand whether KE costs played a role in shaping the monkeys’ trajectory selection (Fig. 6*A*). There were three major steps involved in this modeling. (1) Simulation of trajectories: hypothetical hand and joint trajectories with different levels of deviation were generated using a custom-written function that can generate straight or curved paths between any given pair of points in either space. Curved trajectories were computed using parabolic equations adjusted to pass through the two specified endpoints. Their deviations were controlled by the parameters of the parabolic equations. (2) Transformation of hand trajectories to joint trajectories (this step was only required for simulated hand trajectories): in this step the simulated hand trajectories, defined in Cartesian coordinates, were converted into joint space coordinates (angles of the shoulder and elbow joints). This was done by determining the joint angles required for the KINARM robotic arm to achieve the desired hand positions. Specifically, the KINARM system computes the hand position (*X_h_*, *Y_h_*) from joint angles (*θ_s_*, *θ_e_*) using the following geometric relationships, derived from the configuration file of the exoskeleton:

$$X_{h}-X_{s}=L2\cos\;(\theta_{e}-\theta_{s})+L1\cos\;(\theta_{s}),$$

$$Y_{h}-Y_{s}=L2\sin\;(\theta_{e}-\theta_{s})+L1\sin\;(\theta_{s}).$$

Here $\left(X_{h},Y_{h}\right)$ and $\left(X_{s},Y_{s}\right)$ are the positions of the hand (*x*, *y*) and shoulder (*x*, *y*) in the 2D Cartesian space, respectively. *L*1 and *L*2 are the upper and lower arm lengths, respectively, and $\theta_{e}$ and $\theta_{s}$ are the elbow and shoulder angles, respectively. In our KE estimation model, the joint angles corresponding to a given hand position were obtained by inversely solving these equations. (3) Computation of KE: a time profile was applied to each simulated (or transformed) hand and joint trajectory such that the time intervals between the 501 sample points followed an inverted Gaussian curve. This was done to ensure that the simulated trajectories have bell-shaped velocity profiles mimicking natural movements (Abend et al., 1982). The inverted Gaussian [t(k); Eq. 4] for time intervals was generated by subtracting a Gaussian function [g(k); Eq. 5] from a constant C and then was normalized by the summation of itself to have a total duration of 1 s (the average duration for movements for the monkeys was ∼840 ms):

t(k)=[C−g(k)]/∑(C−g(k)),

$$g(k)=\exp\;\left(-0.5{\left(\frac{k-\mu }{\sigma }\right)}^{2}\right),$$

where k is the sample index (500 time intervals for 501 trajectory points), μ is the midpoint of the samples (250), σ is the width or standard deviation of the Gaussian (75), and C was set to max(g(k))+0.1 to have a positive and nonzero t(k). Since in our analysis we focused on the relative changes in KE across different deviations for each submovement rather than precise velocity contributions, we did not use individually tailored time profiles and used the same time profile for all submovements. The simulated/transformed joint angle trajectories together with the time profile were used to compute the joint velocities (*ω_s_* and *ω_e_*) for every trajectory, which were further used to compute KE following Equation 1.

#### Model validation

For validating the hand-to-joint trajectory transformation and the use of a single time profile in our KE estimation model, we used the hand trajectories from a representative session of Monkey E after replacing their inherent temporal profile with the temporal profile described above. This removed their inherent velocity profile but preserved their shapes. We then transformed the hand trajectories to joint trajectories and computed their KEs as mentioned above. These model-estimated KE for the subtrajectories were then compared with the KE calculated from the recorded joint angular velocities to see if the modeled and observed KE values matched (see Results and Fig. 6*B*).

#### KE estimation for different deviations

For every submovement, KE was estimated for trajectories that had 32 levels of deviation around the straight trajectory. In order to simulate the variability in the monkeys’ hand position at the start and end of the movement, 100 trajectories were simulated at every level of deviation such that the “start” and “end” targets for each trajectory were randomly selected within the 1 cm radius of the target locations. The mean KE of these trajectories (in either space) was then used as the estimated KE for each combination of submovement and deviation.

#### Effective deviation range

**Figure 7.**
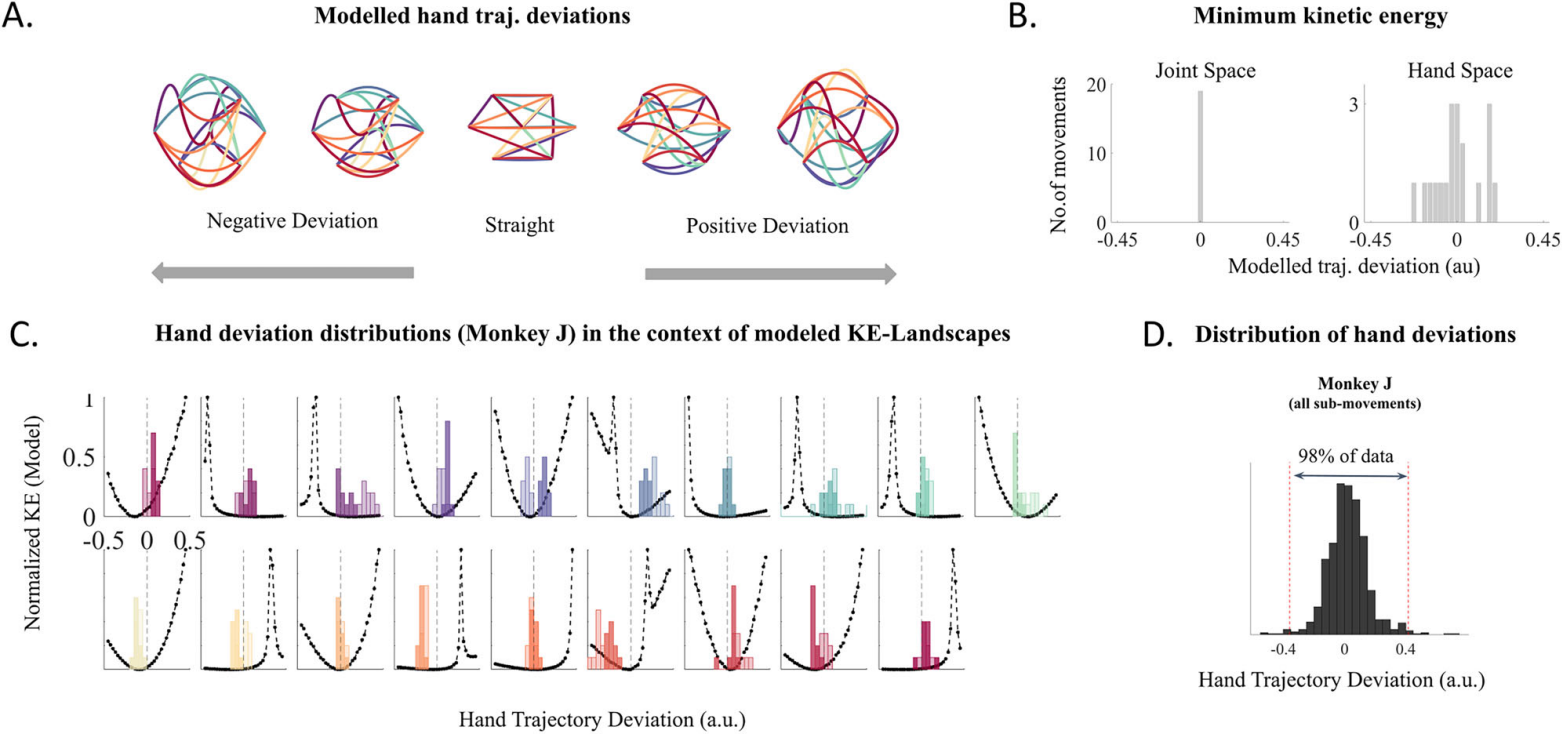
Modeled KE-LS in relation to observed trajectory deviations: ***A***, Simulation of hand trajectories with different levels of deviations, Simulated hand trajectories for all task-relevant submovements for five varying levels of trajectory deviations (left, negative deviations; center, no deviation; right, positive deviations). ***B***, Distribution of deviations showing minimum modeled KE across submovements, Bars indicate the number of submovements that achieve minimum modeled KE for the 33 different modeled deviation levels (left, joint space; right, hand space). ***C***, Observed hand trajectory deviations with respect to modeled KE-LS, Modeled and normalized KE (black dash-dot curve) for 33 different hand trajectory deviations (horizontal axis) are overlaid with the observed distributions of hand trajectory deviations for every submovement (individual subplots). The colors correspond to the color code described in Figure 2*A*. The light and the dark shaded bars show the distribution of hand trajectory deviation in the early and late sessions, respectively. ***D***, Effective hand deviation range (Monkey J), The global distribution of hand deviations for Monkey J is shown by the black histogram. The effective hand deviation range for Monkey J is identified as the range that spans 98% of the central distribution (between the two red lines).

The effective deviation range was defined for each monkey as the central 98% range of the hand trajectory deviations that the monkey produced across all submovements. For Monkey J, this range is −0.4 to 0.4 a.u. (Fig. 7*D*), and for Monkey E, it is −0.25 to 0.25 a.u. This range was used as a reference to ensure that the KE-Landscape (KE-LS) prediction approach remained within behaviorally relevant deviations naturally produced by the monkeys.

#### Prediction of optimal trajectory deviations

**Figure 8.**
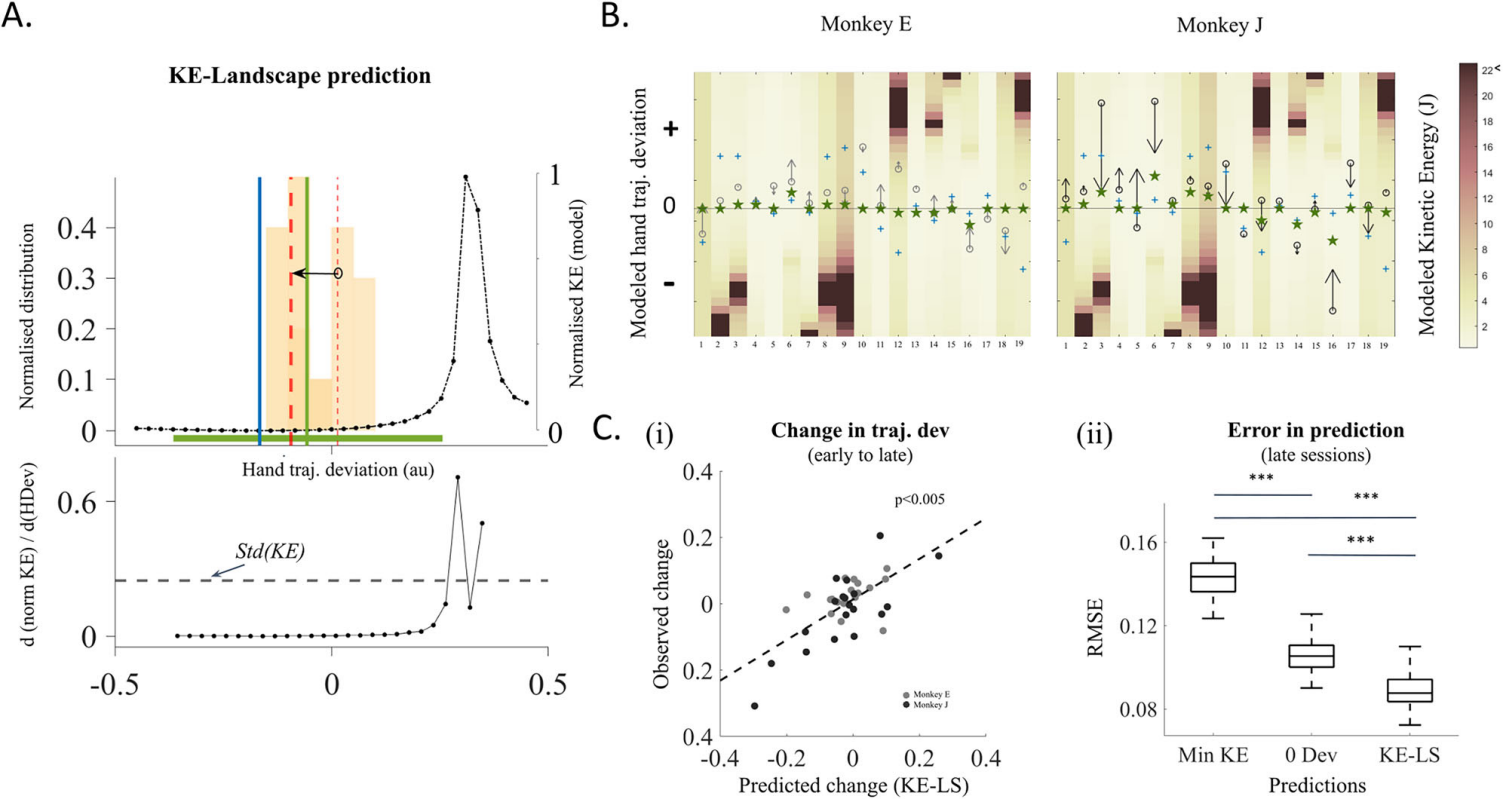
Comparison of observed and predicted evolution of hand trajectory deviations: ***A***, KE-LS prediction (Example Submovement 12, Monkey J). Top, Modeled KE (black dash-dot curve) for 33 different hand trajectory deviations is used to predict the optimal deviation range (green bar). Median deviation (green vertical line) of this range is marked as the KE-LS prediction for this example. The KE minimum is marked by the blue vertical line. For comparison of these predictions with observation, change of hand trajectory deviations from early (light yellow bars) to late sessions (dark yellow bars) is shown by the black arrow. The distribution averages are shown by thin (early) and thick (late) red vertical dashed lines, respectively. Bottom, Unsigned first derivative of modeled KE is shown for deviations spanning Monkey J's effective deviation range (Fig. 7*D*). The dashed horizontal line shows the threshold for the first derivative of KE. ***B***, Evolution of observed trajectory deviations compared with KE-LS predictions. The heatmap shows the estimated KE, each rectangle shows the KE for each submovement (columns) and for 33 deviation levels used in the model. Colors in the heatmap correspond to the color bar on the right (colors saturated to dark brown for KE ≥ 22J). The KE-LS prediction for every submovement using the KE-LS model is marked by a green star (left, Monkey E; right, Monkey J). The Min KE predictions are shown by blue plus signs. The evolution of average trajectory deviation for the monkeys is shown by black arrows (tail, early sessions; head, late sessions). ***C***, Comparison between predicted and observed hand trajectory deviations. ***i***, Comparison of KE-LS model predicted change in average hand trajectory deviation the observed change in average hand trajectory deviation for the monkeys (gray circles, Monkey E; black circles, Monkey J) for each submovement (*p* = 1.2116 × 10^−6^, Pearson's correlation). ***ii***, Comparison of RMSE between Min KE predictions (left), minimum deviation predictions (middle), and KE-LS predictions (right) with respect to observed deviations in late monkey sessions (two-sample *t* test, ***p < 0.0005).

Our model was used to compare the accuracy of three different predictive approaches. (1) KE-LS prediction: an optimal hand deviation range was defined based on the overall relationship between the modeled KE and the effective deviation range as follows. First, for each submovement, the standard deviation of the KEs within the effective deviation range was computed to quantify the typical KE fluctuations in naturally occurring deviations. This standard deviation value was then used to establish a threshold for the absolute first derivative of KE with respect to hand deviations, identifying regions where KE variations are minimal (Fig. 8*A*, bottom). Second, the longest stretch of hand deviations which falls below this threshold was identified. This stretch of hand deviations was considered as the optimal deviation range (or the “KE safe range”), as it represents a region where KE variations remain within a stable range based on the computed threshold (Fig. 8*A*, top, green horizontal bar). Finally, the median of this range was defined as the KE-LS prediction for the given submovement (Fig. 8*A*, top, green vertical line). (2) Minimum KE (Min KE) prediction: the hand deviation corresponding to the minimum modeled KE for each submovement was defined as the Min KE prediction (Fig. 8*A*, top, blue vertical line). (3) Minimum hand deviation prediction: this was always defined by the straight hand trajectory for every submovement.

#### Comparison of prediction accuracies

The accuracy of each prediction method was evaluated by calculating the root mean squared error (RMSE) between the predicted and observed hand deviations in the late sessions (the last 10 sessions for each monkey). To ensure robustness and minimize variability effects, we performed a resampling procedure: the set of submovements was randomly divided into five equally sized subsets (folds), RMSE was computed on each subset, and this process was repeated 20 times with different random splits. The final RMSE values were averaged across repetitions to obtain a stable estimate, reducing potential biases due to data variability.

#### Detection of the movement classes based on trajectory deviations (*K*-means clustering)

A *k*-means clustering algorithm (MATLAB kmeans function) was used to identify the two different submovement classes in terms of the ratio between the hand and joint trajectory deviation. This was done by specifying the distance metric in the MATLAB kmeans function to be the “cosine” between the hand and joint deviations for each submovement across sessions.

#### Statistical reporting

All statistical analyses performed in the study are summarized in Table 1, following the journal's guidelines. Each statistical test reported in the Results section is indexed with a lowercase superscript letter (a–o) corresponding to a specific row in the table. For each test, we provide the type of analysis, the sample size and definition (e.g., number of sessions, trials, submovements, or animals), the exact *p* value, and the 95% confidence interval (CI) of the relevant summary statistic. For nonparametric tests, 95% CI for medians were estimated using bootstrap resampling. For parametric tests (e.g., two-sample *t* tests), the CI for the mean difference was calculated using the standard error and the critical *t* value.

**Table 1.** Statistical table

	Data structure	Type of test	Power
a	Non-normal distribution	Wilcoxon rank-sum test	95% CI: −0.1977 to −0.0688
b	Non-normal distribution	Wilcoxon rank-sum test	95% CI: −0.5117 to −0.1326
c	Non-normal distribution	Wilcoxon rank-sum test	95% CI: −0.0130 to 0.0752
d	Non-normal distribution	Wilcoxon rank-sum test	95% CI: −0.0982 to 0.0107
e	Non-normal distribution	Wilcoxon signed-rank test	95% CI: −0.0066 to 0.0171
f	Non-normal distribution	Wilcoxon signed-rank test	95% CI: −0.0190 to 0.0204
g	Non-normal distribution	Wilcoxon rank-sum test	95% CI: −0.1433 to −0.0263
h	Non-normal distribution	Wilcoxon rank-sum test	95% CI: −0.4465 to −0.1323
i	Non-normal distribution	Wilcoxon rank-sum test	95% CI: −0.0264 to 0.0303
j	Non-normal distribution	Wilcoxon rank-sum test	95% CI: −0.1194 to 0.0101
k	Non-normal distribution	Wilcoxon rank-sum test	95% CI: 1.0589 to 1.2659
l	Non-normal distribution	Wilcoxon signed-rank test	95% CI: −0.1791 to −0.0475
m	Normal distribution	Two-sample *t* test	95% CI: −0.0394 to −0.0338
n	Normal distribution	Two-sample *t* test	95% CI: −0.0543 to −0.0489
o	Normal distribution	Two-sample *t* test	95% CI: −0.0173 to −0.0128

This table summarizes the details of all statistical analyses performed in the study. For each statistical result reported in the manuscript, a corresponding lowercase letter (a–o) is used as a superscript in the Results section to refer to this table. Each row specifies the type of test performed, the population size and definition, the exact *p* value, and the 95% CI for the relevant summary statistic.

#### Code accessibility

The codes described in the paper (KE estimation model and the subsequent analyses) are freely available online at https://github.com/JanaShrabasti/KE_estimation_model.

## Results

### Behavior and example trajectories

The main goal of this study is to provide an in-depth investigation into the factors influencing the acquisition of specific trajectories for 2D visually guided reaching movements in a constrained biomechanical context. Two rhesus monkeys took part in the experiment, utilizing their right arms to perform a sequential reaching task. The setup and the trial structure of this task are depicted in Figure 1 (see also De Haan et al., 2018). The monkeys’ right arms were securely positioned within a robotic exoskeleton (constrained on a horizontal plane) that measured the hand endpoint and joint kinematics at a frequency of 1 kHz (Fig. 1*A*). In each trial, the monkeys had to perform a sequence of movements that started from a central position and involved three consecutive submovements (Fig. 1*B*). Figure 1*C* illustrates the eight distinct movement sequences, repeatedly performed by the monkeys over several weeks of training (insets) and the actual performance of one monkey across repeated trials in a typical training session. These eight movement sequences were composed of 19 different submovements identified by a unique pair of start and end targets. These submovements varied in spatial origins, amplitudes, and orientations and were treated as separate entities in all the following analyses. The trajectories made by the monkeys for each individual submovement reveal specific patterns of trajectory shapes. All the following analyses aim at explaining the factors leading to these typical trajectory shapes and their evolution over multiple sessions.

### Trajectory deviation in hand space and joint space

The 19 submovements included in the eight movement sequences are illustrated in Figure 2*A*, where they are sorted according to their directions in the Cartesian space. A unique cyclic color code is assigned to each submovement and commonly used in all following figures. According to the color code, submovements at the two extremes of Figure 2*A* are represented by very similar colors because they are both in the forward direction. Visual inspection of trajectories both in hand space (with Cartesian coordinates representing the hand position; Fig. 2*B*) and joint space (with angular coordinates representing elbow and shoulder joint angles; Fig. 2*C*) unveils distinct trajectory deviations from a straight line for each submovement. The circles along the courses of the submovements mark the time-resolved standard deviation of trajectories (in a session) around the mean trajectory for every submovement (Fig. 2*D*). For later analyses, the extent of trajectory deviation for each repetition of a submovement was computed as the maximum perpendicular distance of that individual trajectory from the straight line connecting the trajectory's endpoints, normalized by the length (l) of the straight line (Fig. 2*E*).

### Evolution of submovement trajectories across sessions

To investigate how practice affects trajectory deviation, we compared the trajectories of the same submovement across training sessions spanning several months. The evolution of trajectories in the hand space is shown in Figure 3 for three example submovements. The first example (top row) shows very limited changes in deviation of the trajectory in hand space. The second example (middle row) shows striking and nonmonotonic changes in the trajectory shape, from a nearly straight average trajectory in early sessions through a highly deviated average trajectory in intermediate sessions to a moderately deviated average trajectory in late sessions. The final example (bottom row) shows a consistent decrease in deviation of the trajectories over the sessions. The varied evolution patterns of the trajectory deviations for the 19 submovements have been exploited in further analyses to identify which movement parameters are adjusted in priority by the monkeys during practice.

### Is there a common planning space for all submovements?

One possible factor determining the characteristics of the submovement trajectories is the existence of a common space, either hand or joint space, in which all movements are planned. In this case, trajectory deviation for all submovements performed by the monkeys should be restricted to relatively low values in the planning space (either joint or hand space) with limited variability across trials and sessions. In contrast, the deviation in the other space would distribute more widely and reach higher values (Fig. 4*Ai*,*ii*). Our findings did not agree with either of these two predictions. We found that for neither of the two spaces, trajectory deviations were restricted to low values for all the submovements (Fig. 4*B*), and the variability of trajectory deviations (shaded ellipses, color-coded for individual submovement in Fig. 4*B*) captured across sessions was not constrained exclusively to either the hand or joint space axis. There was a strong correlation between the hand and joint trajectory deviations across all the submovements for both monkeys [Pearson's correlation statistics, [*R*, *p*] = [0.6425, 1.26 × 10^−45^] and [0.8253, 8.74 × 10^−20^] for Monkeys E (*N* = 380) and J (*N* = 798), respectively]. The deviations shown in Figure 4*B* were computed across all sessions. As such, they reflect the temporal evolution of these deviations throughout the training period. This analysis approach was intentional: our goal was to evaluate whether trajectory deviations remained consistently more constrained in a specific planning space over time, thus supporting the existence of a common space for movement planning.

The correlation in Figure 4*B* should not be interpreted as implying that hand and joint deviations are perfectly coordinated since some submovements can show similar levels of trajectory deviations in joint space while showing very distinct trajectory deviations in hand space. For instance, a movement that involves only shoulder joint rotation, with a fixed elbow angle, exhibits a straight trajectory in joint space, while the corresponding hand trajectory is an arc of a circle with its center at the position of the shoulder. Conversely, perfect coordination between the two joints will also lead to a straight joint trajectory but a hand space trajectory with a lesser deviation than the previous case.

We confirmed the results of no preference for straight trajectories in either of the two spaces by analyzing trial-by-trial variability within sessions (Extended Data Fig. 4-1), which showed that the principal axes of variability were not consistently distributed to be aligned with either the joint space (90°) or hand space (0°) axis for individual sessions and submovements.

We further investigated the evolution of the trajectory deviations for the submovements in both hand and joint space to see if there is any preference of either of the spaces as a planning space. The existence of a common planning space should logically lead to trajectory optimization, yielding reduced deviations in that space as practice accumulates over sessions (Fig. 5*A*). However, we observed that there was no consistent pattern of reduced trajectory deviation in the later sessions compared with the earlier ones within either the hand (Fig. 5*Bi*, *p_e_* = 0.8675; *N* = 38; Wilcoxon signed-rank test) or joint space (Fig. 5*Bii*, *p_f_* = 0.8790; *N* = 38, Wilcoxon signed-rank test). On the contrary, we observed in each of the two spaces a mixture of trajectories with increasing or decreasing curvatures across sessions. Thus, we do not find any evidence of a common planning space that can explain the trajectory deviation of the submovements.

The lack of evidence for a common planning space for all submovements led us to consider alternative planning criteria and in particular the minimization of specific cost functions such as hand jerk, joint torque, or KE as previously proposed by others (Berret et al., 2011). Our results do not support a universal strategy for minimizing jerk or torque, as we did not observe consistently straight trajectories in either joint of hand space. Indeed, if movement trajectories were planned to minimize jerk, we would expect them to be straight in hand space (Flash and Hogan, 1985), whereas if they were planned to minimize joint torque, they should be straight in joint space (Uno et al., 1989). Instead, our findings prompted us to investigate whether trajectory selection occurs to minimize KE, a widely considered cost function in motor planning (Hatze and Buys, 1977; Nelson, 1983; Cruse, 1986; Soechting et al., 1995; Alexander, 1997; Kashima and Isurugi, 1998; Engelbrecht, 2001; Todorov and Jordan, 2002; Todorov, 2004).

### KE could determine the specific trajectory characteristics

To investigate the effect of KE on trajectory selection, we first examined if there is any KE optimization for the submovements across training sessions. Our analysis revealed a statistically significant general reduction (*p_l_* = 7.7543 × 10^−5^; *N* = 38; Wilcoxon signed-rank test) in the KE during the later sessions compared with the earlier ones across submovements (Fig. 5*Biii*). To further characterize KE evolution at a finer scale, we additionally analyzed the session-by-session progression of KE for each submovement (Extended Data Fig. 5-2*A*). To summarize the direction and consistency of these trends across submovements, we plotted histograms of the Pearson's correlation coefficients (*r*) from the session-wise KE regressions (Extended Data Fig. 5-2*B*). These histograms indicate that a large majority of submovements show negative *r* values (84% in Monkey J, 68% in Monkey E), confirming a widespread tendency for KE to decrease over time. This analysis confirmed that while KE changes are not strictly monotonic across sessions, there is an overall decreasing trend, particularly for submovements with higher initial KE.

The reduced KE across all submovements in later sessions compared with earlier ones suggests that the monkeys may be selecting specific submovement trajectories that optimize KE over time. However, this interpretation is challenging to validate based solely on experimental observations, as trajectory deviations for each submovement are typically restricted to a limited range. This constraint makes it difficult to assess how choosing a different trajectory deviation than the one observed in the monkeys would impact KE. To gain more insights into the complex relationship between movement trajectories and KE, we built a model that allowed us to estimate the level of KE for a wide range of trajectory deviations.

### KE estimation model for differently deviated trajectories

To estimate the KE expenditure for any movement trajectory within our experimental biomechanical setup, we developed a KE estimation model. This model simulates trajectories with varying deviations and estimates required KE expenditure based on the corresponding changes in joint angles following a stereotypical velocity profile. The principles of our KE estimation model are detailed in Materials and Methods and illustrated in Figure 6*A*.

We validated the model's performance by comparing the average KE measured for each submovement of a representative session of monkey E with the average KE estimated for the corresponding submovements from the model. The model estimated KE for trajectories with an artificial bell-shaped velocity profile that retained the shape as performed by the monkey. The close agreement of the estimated and observed energy values (Fig. 6*B*), along with the highly significant positive correlation (Pearson's correlation, *r* = 0.98; *p* = 3.1 × 10^−14^; *N* = 19) between them, confirms the model's reliability as a KE estimator for any trajectory defined in either the hand space or the joint space, within the context of the current 2D movement setup.

Leveraging this model, we proceeded to estimate KEs associated with simulated trajectories for every submovement at 32 different levels of hand and joint trajectory deviations around the straight trajectory. Figure 7*A* illustrates the modeled hand trajectories for two levels of deviations on both sides of the straight trajectory. By convention, we assigned negative values to deviations on the left of the straight trajectory in the direction of movement and positive values to opposite deviations.

First, for each submovement in joint and hand space, we identified the trajectory deviations requiring the least energy (Fig. 7*B*). As predicted from the KE equation (Fig. 6*A*), straight trajectories in joint space correspond to the Min KE for all submovements (Deviation = 0; Fig. 7*B*, left). In contrast, there seems to be no straightforward relationship between the trajectory deviations in hand space and the Min KE, which corresponds to trajectories with various levels of deviation around the straight line (Fig. 7*B*, right).

### Modeling results to understand the mechanism of KE optimization

In addition to identifying the deviations corresponding to the KE minima, our model also provides KE values associated with a wide range of trajectory deviations, hereafter referred to as the KE-LS, for each submovement. The obtained KE-LS for hand space are shown in Figure 7*C* (black dashed-dotted curves), together with the trajectory deviations performed by Monkey J, for all the submovements (colored bars). Visual inspection of these plots highlights several important features. Firstly, about the KE-LS, the relationship between hand trajectory deviations and their corresponding KE globally follows a nonsymmetrical parabola around a Min KE value, with some irregularities in the form of local peaks in some cases. Based on the inverse kinematic transformation from the model, these peaks can be attributed to hand trajectory deviations involving complex joint coordination patterns. Secondly, in most of the submovements, hand deviations in trials from early sessions (light bars) are distributed on the side of the parabolas with smaller KE variations. Notably, 78% of the submovements had average early hand deviations on the side of the parabola corresponding to smaller KE variations. Interestingly, in the late sessions (dark bars), hand trajectory deviations show some evolution but not very consistently toward either the Min KE or minimum hand deviation. Rather, we find that the late hand deviations evolve to or remain on the side of the parabolas that have smaller KE variations. This led us to consider the possibility that trajectories might be optimized by considering the overall KE-LS. Specifically, this suggests that the trajectories evolve toward a region where variations in trajectory deviations in hand space have minimal impact on KE, rather than precisely targeting the KE minimum. To test this, we used our KE estimation model to predict the trajectory deviation performed by the monkey based on the KE-LS.

The derivation of KE-LS prediction is illustrated in Figure 8*A* for an example submovement (Submovement 12, Monkey J). For this, we first defined an optimal range of hand trajectory deviations, where changes in KE between neighboring hand deviations were within the standard variability of the modeled KE for this submovement. This optimal hand deviation range was derived based on the slope of the KE-LS. The standard deviation of the estimated KE values in the central 98% of the hand deviation distribution for each monkey (Fig. 7*D*, effective deviation range for Monkey J) served as the threshold for the slope of the KE-LS, i.e., the absolute first derivative of modeled KE (Fig. 8*A*, bottom). The rationale behind using the standard deviation of the KE within the effective deviation range is that the standard deviation represents the typical variability of KE for a particular submovement within its effective deviation range. If the derivative of KE with respect to hand trajectory deviation (i.e., the change of KE in unit hand trajectory deviation) is smaller than this threshold, then deviations within that range lead to KE changes that are not meaningfully different from the typical modeled KE variation. Thus, these regions of the energy landscape are effectively flat or plateau-like and can be interpreted as energetically comparable from the system's point of view. We identified the optimal deviation range (Fig. 8*A*, top, green bar) as the longest continuous stretch of deviations in the hand space where the absolute first derivatives of modeled KE with respect to trajectory deviations were consistently below this threshold. The median of this deviation range is marked as the KE-LS prediction (Fig. 8*A*, top, green vertical line). In this example, we observe that the average trajectory deviation of the late sessions (bold red dashed line) is very close to the KE-LS prediction, which is distinct from both the KE minimum (blue vertical line) and the straight trajectory (0 deviation).

We evaluated if these observations hold true across all the other submovements, for the two monkeys. The modeled and observed data for all submovements are summarized in Figure 8*B* for Monkey E (left plot) and Monkey J (right plot). In these plots, the modeled KE levels are color-coded according to the color scale on the right, with each column representing a submovement and each row representing 1 of the 32 deviation levels around the straight trajectory in hand space (central horizontal line). Blue pluses mark the hand deviations corresponding to the Min KE of each submovement. The KE-LS predictions, computed as shown in Figure 8*A*, are indicated by green stars for each submovement. These predicted deviations can be compared with the evolution of the mean trajectory deviation between early and late sessions in the observed data, as represented by the arrows. For each submovement, the arrow tail (black circle) indicates the mean trajectory deviation in early sessions, and the arrowhead indicates the mean deviation in late sessions. From this figure, we can observe that many of the arrow heads lie very close to or point toward the direction of the KE-LS predictions. We compared the predicted and observed changes in the hand trajectory deviations for all submovements in the two monkeys. Here, the “predicted” change refers to the difference between the early-session median hand trajectory deviation and the median of the KE-LS prediction for each submovement. The strong correlation between predicted and observed changes in trajectory deviations (Fig. 8*Ci*, Pearson's correlation, *r* = 0.6962; *p* = 1.2116 × 10^−6^; slope = 0.62; *N* = 38) indicates that the monkeys adjust their hand trajectory deviations over sessions to approach the predicted optimal range derived from the KE-LS.

We compared the accuracy of these KE-LS predictions with the predictions obtained using two alternative approaches: (1) an approach based on the principle of minimization of KE, in which the predicted deviation is the hand trajectory corresponding to the Min KE and therefore a straight trajectory in joint space and (2) an approach based on the principle of minimization of hand trajectory deviation, in which the predicted deviation corresponds to the straight trajectories in hand space (deviation = 0). The RMSE of these three predictions compared with observed deviations in the monkeys’ last 10 sessions were calculated using a resampling procedure to ensure robustness and minimize variability effects. Specifically, we randomly split the set of submovements into fivefold, computed RMSE for each fold, and repeated this process 20 times. This approach allowed us to assess the consistency of RMSE estimates and reduce potential biases due to the limited number of submovements. The RMSEs for the Min KE predictions and minimum hand trajectory deviation predictions (0 Dev) are significantly larger than those for the KE-LS predictions [Fig. 8*Cii*, right, comparison of RMSE between different models (*N* = 100) through a two-sample *t* test, *p_m_* = 2.7431 × 10^−65^ between Min KE and 0 Dev; *p_n_* = 1.5954 × 10^−92^ between Min KE and KE-LS; and *p_o_* = 2.4295 × 10^−28^ between 0 Dev and KE-LS].

These results suggest that in the complex biomechanical context of our task, monkeys perform each movement with typical trajectory deviations that are confined to a deviation range corresponding to a “safe KE range.” This safe KE range is characterized by minimal changes in KEs for neighboring trajectory deviations. This finding justifies the observed influences of KE over trajectory deviation patterns observed in many studies including the present one. Additionally, it also provides explanations as to why in certain cases trajectories may not converge to a deviation corresponding to an erratic KE-LS even if it globally could correspond to the energy minimum.

## Discussion

In this study, we investigated the factors influencing the acquisition and evolution of specific trajectories for 2D visually guided reaching movements in monkeys. Unlike previous studies with humans performing movements in a single session (Morasso, 1981, 1983; Abend et al., 1982; Nelson, 1983; Hogan, 1984; Atkeson and Hollerbach, 1985; Flash and Hogan, 1985; Cruse, 1986; Cruse and Brüwer, 1987; Hogan et al., 1987; Hogan and Flash, 1987; Uno et al., 1989; Rosenbaum et al., 1995; Soechting et al., 1995; Haggard and Richardson, 1996; Alexander, 1997; Kashima and Isurugi, 1998; Rasmussen et al., 2001; Bongers and Zaal, 2010; Kistemaker et al., 2010), our extensive dataset from two monkeys across multiple sessions allowed us to identify the movement parameters that are prioritized and optimized through changes in movement trajectories over practice.

By examining trajectory deviations in both hand and joint spaces, we first found the absence of a common planning space for all submovements performed by the monkeys. Specifically, neither hand nor joint spaces show trajectories that tend to be straight for every submovement, and no significant reduction in trajectory deviations is found over the course of practice in either space. These observations may explain the inconsistencies in previous studies attempting to identify a common planning space (Morasso, 1981; Abend et al., 1982; Hogan, 1984; Atkeson and Hollerbach, 1985; Flash and Hogan, 1985; Hogan and Flash, 1987; Uno et al., 1989; Rosenbaum et al., 1995; Haggard and Richardson, 1996; Malfait et al., 2002).

The lack of a common planning space and the distinct patterns of trajectory deviations in hand and joint space across submovements prompted us to investigate alternative trajectory optimization strategies. Based on a body of previous work (Hatze and Buys, 1977; Nelson, 1983; Cruse, 1986; Soechting et al., 1995; Alexander, 1997; Kashima and Isurugi, 1998; Engelbrecht, 2001; Todorov and Jordan, 2002; Todorov, 2004), we explored the assumption that the level of KE is the critical factor determining the selection of trajectory deviations. In support of this hypothesis, we observe an overall reduction in KE over training sessions across submovements, indicating adaptive adjustments in movement strategies to optimize KE expenditure.

Based on this observation, one of the main expectations regarding trajectory selection is that deviations are selected to avoid trajectories requiring high levels of KE. This hypothesis is however difficult to confirm using the experimental data in which both naturally observed deviations and the corresponding KE for each submovement remain within a limited range for each monkey and do not cover the full spectrum of potential trajectories. Additionally, if the naturally observed trajectory deviations are indeed constrained by the KE level, it is unclear whether the motor system adjusts the trajectory deviations to achieve the Min KE, as conventionally thought (Hatze and Buys, 1977; Rasmussen et al., 2001), or if KE is optimized in a different manner that does n't necessarily aim at reaching the Min KE trajectories. The latter hypothesis aligns with previous findings showing that subjects do not always execute lower energy trajectories (Kistemaker et al., 2010).

To address these alternative hypotheses, we used a simple inverse-kinematic model (KE estimation model) to investigate the relationship between trajectory deviations and KE. This model allowed us to examine modulations of KE over a wide range of trajectory deviations, extending beyond those naturally performed by the monkeys for individual submovements. Through this approach, we first confirmed that straight joint trajectories correspond to Min KE values for every submovement, whereas Min KE values may also correspond to curved trajectories in the hand space. These observations are consistent with the predictions of studies addressing questions related to hand trajectory deviations in a constrained 2D setup (Hogan et al., 1987; Uno et al., 1989). Second, we observed that the KE-LS for a wide range of hand trajectory deviations have a nonsymmetrical parabolic shape. We gained interesting insights from the comparison of these KE-LS and the hand trajectory deviation distributions from early and late sessions from the monkeys.

We found that the trajectory deviations performed by the monkeys, particularly in early sessions, are systematically distributed on the shallower side of the KE-LS for most submovements. Based on this, we predicted optimal deviation ranges where changes in trajectory deviation have minimal effects on KE. The KE-LS predictions match well with the evolution of trajectory deviations observed in the monkeys. Comparing predicted hand trajectory deviations with those observed in late sessions, we also found that the KE-LS predictions are more accurate than predictions based on conventional approaches like minimizing KE or trajectory deviation in hand space. To our knowledge, no other study has used modeled KE for a wide range of deviations to predict an optimal range of hand trajectory deviations where a change in trajectory deviations has minimal impact on KE. Our novel approach demonstrates that in a biomechanical context similar to ours, the monkeys’ motor systems optimize movement trajectories not by aiming for precise deviations corresponding to maximum straightness or Min KE but by targeting a deviation range where some variability will not change the energy requirements of a movement drastically.

In further support of the KE-LS hypothesis, a closer inspection of Figure 4*B* reveals that for Monkey E, submovements can be distinguished into two classes based on their levels of deviation in the joint space. Further analysis showed that these two classes of submovements could be identified using a *k*-means clustering approach (with *k* = 2), which consistently revealed a clear and reproducible structure across monkeys. Although *k*-means will always produce two clusters when *k* = 2 is specified, the resulting classification was notable in that the cluster memberships were identical for both monkeys, despite the clustering being performed independently (Extended Data Fig. 4-2*A*). One submovement class exhibits highly distributed trajectory deviations in both hand and joint spaces, whereas the other class displays low trajectory deviations in joint space but more distributed deviations in hand space. Interestingly, we also found that the submovement class with the lower joint space deviations had a higher average KE (Extended Data Fig. 4-2*B*) and had less variable changes of KE with practice (Extended Data Fig. 4-2*C*). Taken together, these complementary results suggest that the way trajectory deviations are distributed in hand and joint spaces is shaped by submovement-specific KE constraints. The KE-LS modeling indicates that KE imposes limits on how trajectories can vary, effectively constraining the allowable deviation ranges in each space. The *k*-means clustering further reveals that submovements naturally group into two classes with distinct deviation patterns, and this structure also systematically reflects their KE levels: submovements with higher KE tend to show constrained deviations in joint space, while those with lower KE show broader joint space variability. Future experiments with submovements defined a priori on the basis of the KE levels would be required to validate these observations.

Even though the KE-LS predictions have lower errors for deviations in late sessions than the minimum hand deviation predictions, we find some evidence of the effect of visual feedback on defining hand trajectories. We selectively looked at the change in trajectory deviations across sessions for submovements with high early deviations in hand space (Extended Data Fig. 5-1). We found that there is a significant decrease in hand trajectory deviations for these movements but no significant reduction in joint trajectory deviations. This suggests that the motor system possibly reduces hand trajectory deviations for submovements that start with a high hand trajectory deviation, without prioritizing reduction of joint trajectory deviations (or KE). Even though this result points at the influence of visual feedback on trajectory selection, as suggested by previous studies (Kistemaker et al., 2010; Vaidyanathan et al., 2020), in our current study it was difficult to completely disentangle the effect of KE and visual feedback, because for a lot of submovements the low KE zones of the KE-LS lay in close proximity to the straight trajectories.

While our findings contribute to a deeper understanding of the interplay between energetic considerations and motor control in a 2D movement setup, certain limitations should be acknowledged. First, we quantified trajectory deviations using the maximum distance from a straight-line trajectory. While this measure provides an intuitive and direct assessment of trajectory straightness, it reduces an entire movement path to a single value, potentially overlooking finer variations in curvature. Alternative measures, such as curvature (*κ*), commonly used in whisker movement analyses (Luo and Hartmann, 2023), could capture local variations in trajectory shape more comprehensively. Future work could incorporate such measures to refine our understanding of trajectory selection.

Second, our findings are based on 2D planar movements in an exoskeletal setup, which significantly constrains hand movements. While this setup allowed for precise control over hand space and joint space kinematics, it remains unclear how our results would generalize to naturalistic 3D movements, where additional degrees of freedom may influence trajectory variability.

Finally, although this study provides valuable insights into the biomechanical constraints underlying motor performance, a more comprehensive understanding of trajectory selection requires further investigation into the neural mechanisms involved in trajectory planning. Future studies of neural processes in motor and premotor areas could help to understand if motor planning integrates the biomechanical constraints of movements to plan the corresponding trajectories.

## Synthesis

Reviewing Editor: Nicholas J. Priebe, The University of Texas at Austin Decisions are customarily a result of the Reviewing Editor and the peer reviewers coming together and discussing their recommendations until a consensus is reached. When revisions are invited, a fact-based synthesis statement explaining their decision and outlining what is needed to prepare a revision will be listed below. The following reviewer(s) agreed to reveal their identity: Mark Humphries. Note: If this manuscript was transferred from JNeurosci and a decision was made to accept the manuscript without peer review, a brief statement to this effect will instead be what is listed below.

Two reviewers evaluated the manuscript "Energy constraints determine the selection of reaching movement trajectories in macaque monkeys." The manuscript examines the evolution of monkeys' arm movement trajectories over sessions in a 2D sequential target task, and shows evidence that trajectories evolve to minimize the kinetic energy cost of deviations in the trajectory. The reviewers appreciated the approach of the manuscript, but also raised a number of concerns with the analysis of the data. In particular, reviewers raised the question of whether the clustering into two movement subclasses was appropriate. It was not clear that this clustering was statistically justified or if it is necessary for the arguments presented in the manuscript. This issue and others are detailed below in both reviewers' comments which should be useful in revising your manuscript, if you decide to resubmit it to eNeuro or to another journal.

Reviewer #1

1. This article examines the criteria being used for movement planning. The authors chose to examine KE, hand paths and joint angles. There are however many other features that have now been considered as planning criteria such as minimum jerk, torque or minimum end point errors. The authors should explain a little why some of these other cost functions were not considered.

2. The transition from Figure 1 to Figure 2 is unclear. In counting the number of trajectories in Figure 1, there are a total of 24 trajectories. In Figure 2, there are 19.

3. It is important in the Methods section to specify the distance between the targets. Perhaps I missed this information?

4. Figure 3 - It would be better to choose more widely varying colors between the left and the right of the color bar. At the two extremes they appear very similar.

5. Using K means the authors have found two clusters. One in which there are big deviations in joint trajectories, and a second in which this is not the case. Were the authors able to identify which types of sub movements were in C1 and which in C2? Was there any consistency in this? Also, while correlation between hand and joint trajectories is used as a distance measure, there is no mention of the differences in this variable as the authors characterize the 2 clusters.

6. I have difficulty with Figure 4 - I am not sure that the figures 4A i and ii are necessary. I am unsure of what 4A iii indicates. It appears to indicate the type of relationship which should exist between early and late trajectory deviations, if there is a common planning space between hand and joint space trajectories. But, looking at the legends for the figure, I do not think this is the case.

7. Figure 4B appears to lack some important quantification. Given the small number of points, it appears that the authors are using average deviation across several trials. Instead it would be interesting to see the values of correlations present between hand and joint deviations on a trial by trial basis. Significant correlations or lack thereof in these trial by trial deviations should be a better indicator of common planning space. This makes of course a lot of trials, but perhaps the authors could break it down into C1 and C2 trajectories?

8. The authors state that decreasing KE with successive trials, would support the argument that some movement planning takes place in this space. Once again, the quantification on this question in the current paper does not always appear straightforward. What does Figure 5 ii add to this question? Wouldn't a trial by trial quantification of normalized (to take into account original values) change in KE energy over sessions, give a clearer indication of increase or decrease in KE over trials? Some quantifications of trends in KE over successive trials should indicate how KE progresses over time. Why instead is there a coarse division into early and late sessions and seen in Figure 5Bii?

9. The authors have based their conclusions on the safe KE range based on a model using movements which were not performed by the monkey. It might be useful to clearly indicate where the natural movements falls so that it is easy to verify if such KE regulation also held for the real movements. If not, another figure with only the real movements might be even better.

Reviewer #2

This paper investigates the evolution of monkeys' arm movement trajectories over sessions in a 2D sequential target task, and shows evidence that trajectories evolve to minimise the kinetic energy cost of deviations in the trajectory. This hypothesis better correlates with the changes in trajectories across sessions than minimising the kinetic energy or minimising hand deviation from a straight line. There is also some interesting evidence here against the hypotheses of a single joint-centric or hand-centric movement planning space.

Major comments:

(1) The paper makes much of the two classes of submovements, but I do not see convincing evidence for them. Lines 364-5 claims observation of "two classes of sub-movements with distinct patterns of trajectory deviation", but I do not see them. It seems from Fig 4B that the two claimed classes are one of sub-movements with a correlation of their deviations in hand-centric and joint-centric space, and one of sub-movements that do not correlate their deviations. There is a suggestion of this for Monkey E; but for Monkey J this just looks like a strong correlation between deviations in both hand and joint space. k-means will of course return two classes because that's what it was asked to do. Convincing evidence would mean showing that there are indeed two classes using an unsupervised clustering approach. Even then it is not clear a priori why two such classes would exist, hence the rationale for looking for classes in the first place is unclear.

But then nor do I see the need for defining any classes either, as none of the main insights from the paper rests on those classes. Indeed, the whole issue seems to obscure what could be stronger evidence for the main claim of their being no hand-centric or joint-centric planning space. Suggest the following:

- ditch the class-based analyses (Fig 4, 5 and end of Figure 8, parts of Discussion).

- Instead, do the key analyses on all 19 sub-movements, not just 2 arbitrary classes. i.e.

(i) Fig 4B: the prediction in Fig 4A is that there should not be any correlation between deviations in hand and joint space. So just report the correlations between them in Fig 4B - they look strong.

(ii) Fig 4C: similarly, this analysis of change in deviation does not need the added complexity of "classes"

(iii) Fig 5: unclear what this figure and Results section are adding to the analysis: Fig 5Aii shows evidence of KE reducing with practice, which foreshadows the findings in Fig 8.

- comment on how there can be deviations in hand space without deviation in joint space: how is this possible given the exoskeleton constraints on arm movements? is this solely wrist changes, or just the limits on hand-centric measurements given different finger positions?

Minor comments:

- Line 477 &Methods: how can the width of a data histogram of hand movement deviations (in a.u.) serve as the threshold for a differential equation of energy? (Fig 8A).

- lines 510-512 and Methods: it is claimed that the RMSE for the different models is obtained by cross-validation. But no models are fit to data here in a "training" fold then evaluated on a "validation" fold: for each sub-movement there is just one data-value for the actual change in hand deviation, and three other values (KE-landscape, KE-min, zero-deviation) to compare it to. It sounds more like this is a resampling analysis, but resampling of what and to compute what?

- Statistics need some work throughout:

(i) Pearson's correlation coefficient is computed, but not reported! Give the R obtained, as this is the measure we need to know to assess the correlation (the p-value just tells us if there is any reason to doubt the correlation value)

(ii) No stats test has its N reported.

- Discussion contains no discussion of limitations. At least comment on:

(a) the use of maximum distance from a straight-line trajectory as the measurement of deviation: this reduces an entire curve to a single number that may not capture the full extent of the deviation. An alternative would be to use a full measure of line curvature i.e. kappa (see eg its routine use in whisker curvature analysis: https://journals.plos.org/plosone/article?id=10.1371/journal.pone.0269210)

(b) how the findings here in 2D planar movements with no rotation of the shoulder may generalise to 3D movements

Text suggestions

- Define KE in the Significance Statement

- line 96: perhaps explain how surface shape and friction can influence arm trajectory in free movement

- Eq 2 and text: where does Eq 2 come from? And how were the moments of inertia calculated?

- Methods: define early/late sessions
