# Supplementary figures and images for: Energy Constraints Determine the Selection of Reaching Movement Trajectories in Macaque Monkeys
Source: eNeuro. 2025 Sep 30;12(10):ENEURO.0385-24.2025. doi: 10.1523/ENEURO.0385-24.2025 (PMC12528833; doi:10.1523/ENEURO.0385-24.2025)

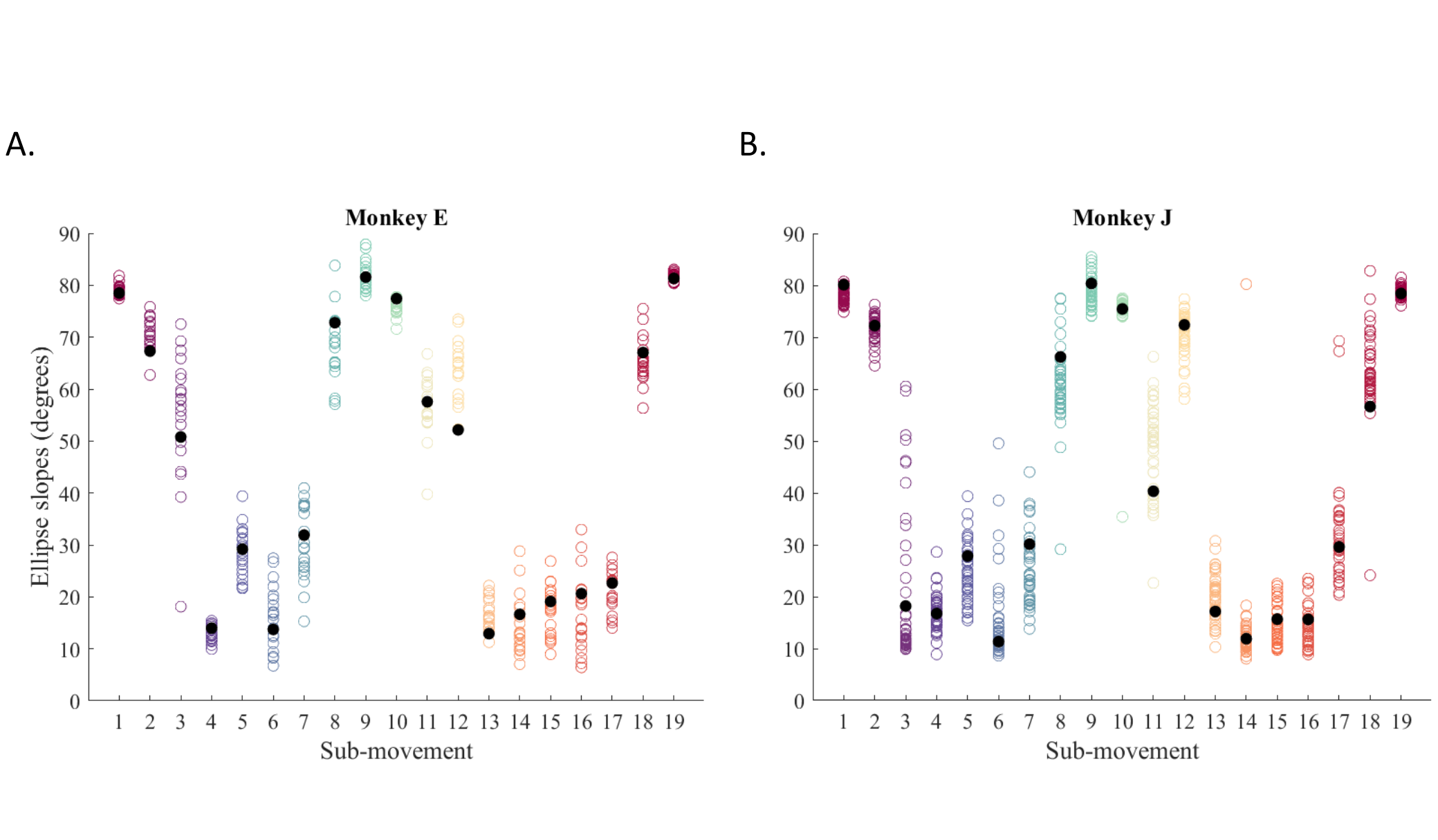

Supplement: Figure 4-1 — Distributions of axis angles of variability ellipse for sub-movements across sessions: For each sub-movement (n = 19), we quantified trial-by-trial variability in hand-space and joint-space deviations within individual sessions. For each session and sub-movement, an ellipse was fitted to the distribution of deviations (similar to Figure 4B but for individual sessions), and the angle between the major axis of the ellipse and the horizontal (hand-space) axis was measured. This angle captures the direction of maximal trial-by-trial variability relative to hand and joint spaces. The distributions of these angles across sessions are plotted here separately for each sub-movement (color-coded according Figure 2). The angles for such ellipses shown in Figure 4B (captures variability of session-averaged deviations across sessions) have also been plotted for comparison and marked by a black circle for each sub-movement. A: Monkey E and B: Monkey J. Download Figure 4-1, TIF file. [file eneuro-12-ENEURO.0385-24.2025-s001.tif]

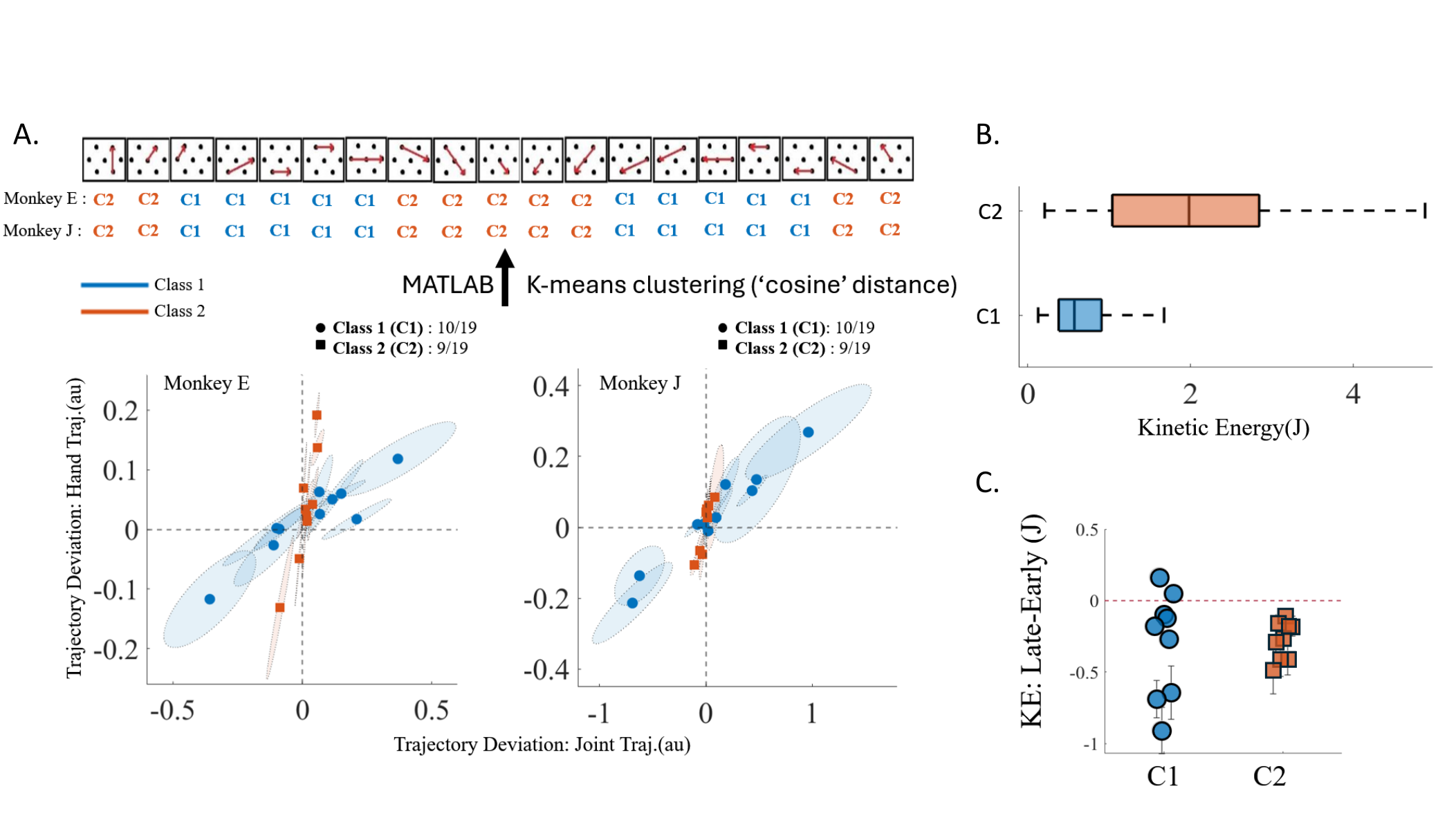

Supplement: Figure 4-2 — Sub-movement classes based on hand and joint deviations: A. Sub-movement classes - Two sub-movement classes (Blue- Class 1, Orange- Class 2) were identified from Figure 4B, using kmeans clustering algorithm (distance parameter - cosine). The sub-movements classified in the two classes were identical for both the monkeys as shown in top panel. B. The boxplots show the KE distribution for sub-movements in classes C1 and C2, with C2 having significantly higher energy than C1 (p = 4.7862×10^-80^, Wilcoxon rank-sum test). C. Circles indicate the magnitude of change in KE for individuals with significant differences between early and late sessions (left - C1; right - C2), with error bars representing standard error. Download Figure 4-2, TIF file. [file eneuro-12-ENEURO.0385-24.2025-s002.tif]

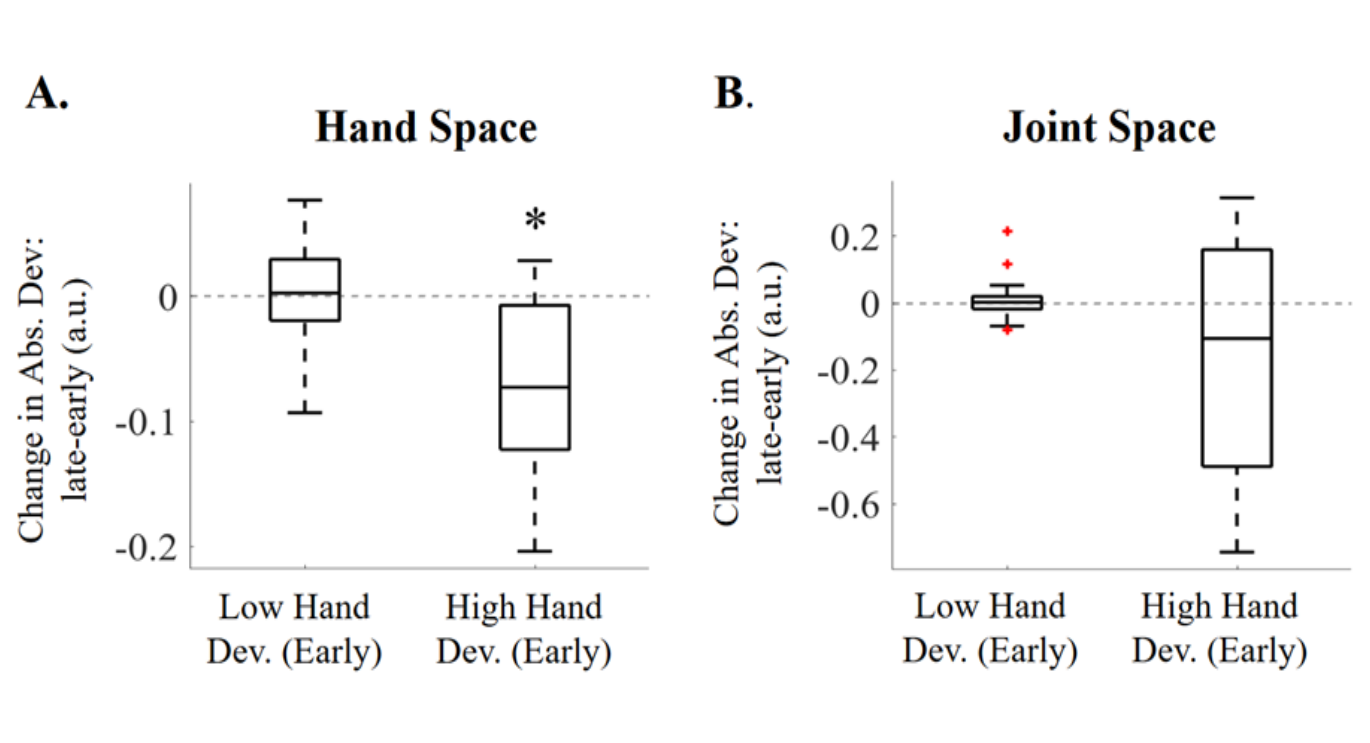

Supplement: Figure 5-1 — Change in deviations for movements with high/low initial hand deviation: The change in absolute trajectory deviation in both hand space (A) and joint space (B) is computed for the movements that have low early hand deviations and high early deviations. Only for movements which had high initial hand deviations, a significant reduction (p<0.05: paired sample t-test) in hand deviation was observed. Download Figure 5-1, TIF file. [file eneuro-12-ENEURO.0385-24.2025-s003.tif]

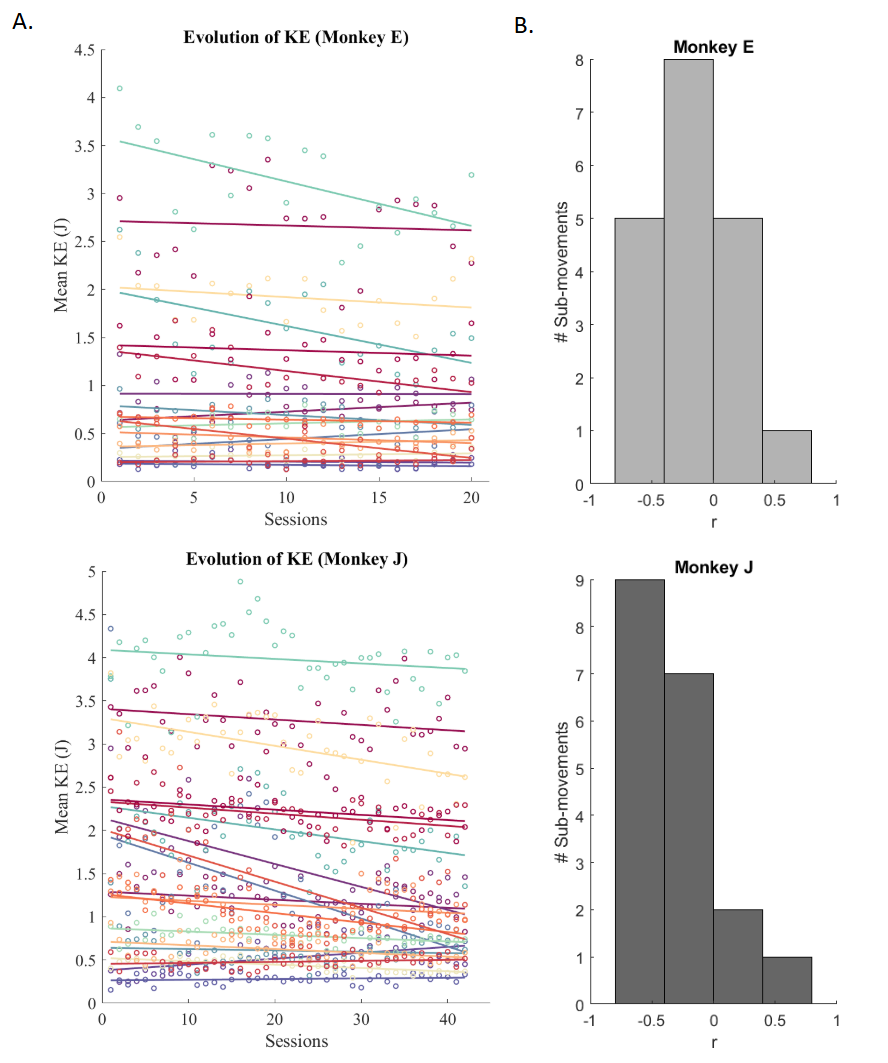

Supplement: Figure 5-2 — Evolution of KE through sessions: A. For each sub-movement, the average KE for each session was calculated and plotted as a open circle. Linear regression lines were fitted to the KE values across sessions to estimate the overall trend of KE changes over time. Each panel shows the session-by-session progression of KE for one monkey. The markers and regression lines corresponding to the 19 different sub-movements are color-coded to the code presented in Figure 2. B. Histograms of Pearson correlation coefficients (r) from the linear regression models for each monkey summarize the consistency and directionality of KE changes across sub-movements. The overall shift of r values toward the negative side indicates a prevalent trend of KE reduction over time. Download Figure 5-2, TIF file. [file eneuro-12-ENEURO.0385-24.2025-s004.tif]
